# Restriction endonucleases that cleave RNA/DNA heteroduplexes bind dsDNA in A-like conformation

**DOI:** 10.1093/nar/gkaa403

**Published:** 2020-05-27

**Authors:** Marlena Kisiala, Monika Kowalska, Michal Pastor, Henryk J Korza, Honorata Czapinska, Matthias Bochtler

**Affiliations:** International Institute of Molecular and Cell Biology, Trojdena 4, 02-109 Warsaw, Poland; Institute of Biochemistry and Biophysics PAS, Pawinskiego 5a, 02-106 Warsaw, Poland; Biological and Chemical Research Centre, University of Warsaw, Zwirki i Wigury 101, 02-089 Warsaw, Poland; International Institute of Molecular and Cell Biology, Trojdena 4, 02-109 Warsaw, Poland; International Institute of Molecular and Cell Biology, Trojdena 4, 02-109 Warsaw, Poland; Institute of Biochemistry and Biophysics PAS, Pawinskiego 5a, 02-106 Warsaw, Poland; Syngenta, Jealott's Hill International Research Centre, Bracknell, Berkshire RG42 6EY, UK; International Institute of Molecular and Cell Biology, Trojdena 4, 02-109 Warsaw, Poland; Institute of Biochemistry and Biophysics PAS, Pawinskiego 5a, 02-106 Warsaw, Poland; International Institute of Molecular and Cell Biology, Trojdena 4, 02-109 Warsaw, Poland; Institute of Biochemistry and Biophysics PAS, Pawinskiego 5a, 02-106 Warsaw, Poland

## Abstract

Restriction endonucleases naturally target DNA duplexes. Systematic screening has identified a small minority of these enzymes that can also cleave RNA/DNA heteroduplexes and that may therefore be useful as tools for RNA biochemistry. We have chosen AvaII (G↓GWCC, where W stands for A or T) as a representative of this group of restriction endonucleases for detailed characterization. Here, we report crystal structures of AvaII alone, in specific complex with partially cleaved dsDNA, and in scanning complex with an RNA/DNA hybrid. The specific complex reveals a novel form of semi-specific dsDNA readout by a hexa-coordinated metal cation, most likely Ca^2+^ or Mg^2+^. Substitutions of residues anchoring this non-catalytic metal ion severely impair DNA binding and cleavage. The dsDNA in the AvaII complex is in the A-like form. This creates space for 2′-OH groups to be accommodated without intra-nucleic acid steric conflicts. PD-(D/E)XK restriction endonucleases of known structure that bind their dsDNA targets in the A-like form cluster into structurally similar groups. Most such enzymes, including some not previously studied in this respect, cleave RNA/DNA heteroduplexes. We conclude that A-form dsDNA binding is a good predictor for RNA/DNA cleavage activity.

## INTRODUCTION

Double-stranded RNA (dsRNA) and RNA/DNA heteroduplexes (hybrids) adopt a conformation close to the A-DNA form. They have a narrower major and a wider minor groove than hydrated dsDNA, which tends to assume a B-form structure (1). As base pair hydrogen bonding patterns are unequivocal only in the major groove (2), cleavage sites in dsRNA or RNA/DNA duplexes tend to be defined by the distance to the nucleic acid terminus or by secondary structure, rather than by nucleotide sequence (3–6). Therefore, sequence-specific endonucleases, directed against either dsRNA or RNA/DNA, are expected to be both rare and difficult to engineer.

Restriction endonucleases are prototypes of enzymes that target dsDNA with exquisite sequence specificity (7). *A priori*, there is no reason to expect that any restriction enzyme may sequence specifically cut dsRNA or RNA/DNA. Optimization for dsDNA should drive substrate binding groves toward accommodating B-form and away from accepting A-form DNA. Moreover, poor accessibility of the major groove makes non-degenerate sequence readout in A-like nucleic acids difficult.

A few years ago, Richard Roberts and co-workers carried out an extensive screen of restriction endonucleases in the hope to find enzymes that could be useful as tools for RNA biology (8). As they expected based on very different structures of dsDNA and dsRNA, none of the tested enzymes could cleave dsRNA in a sequence-specific manner. Surprisingly, some restriction enzymes including AvaII, BanI and TaqI could cleave one or both strands of RNA/DNA heteroduplexes. For practical applications, the most interesting enzymes are those that cleave at least the RNA strand of the heteroduplex, since they may be used together with a ‘helper’ DNA oligonucleotide to cleave RNA at a defined site. This property could be exploited in practice to create RNA molecules with defined ends (as an alternative to the use of ribozymes, DNAzymes or CRISPR enzymes), e.g. for structural studies or splinted ligation of RNA fragments (8).

Unfortunately, the biochemical screening could not clarify why a few restriction endonucleases could cleave RNA/DNA heteroduplexes, whereas most others did not. Almost all of the RNA/DNA cleaving enzymes (PflMI is the exception) are known or predicted to belong to the Type II, PD-(D/E)XK family of restriction enzymes. Type IIS enzymes that cleave at a distance from their recognition site were not represented. Enzymes shown to be active on RNA/DNA heteroduplexes cut within palindromic or nearly palindromic recognition sequences. *A priori*, one could expect cleavage to occur for RNA/DNA heteroduplexes with a tendency to adopt a B-like conformation, which is known to be correlated with a high purine content of the DNA strand (9). However, for palindromic or nearly palindromic sequences, the DNA (and RNA) strand purine content is at or near 50%, so that this effect cannot be decisive.

AvaII from the filamentous cyanobacterium *Anabaena variabilis* has attracted our interest because it exhibits robust activity against the RNA and DNA strands of an RNA/DNA heteroduplex. As most other enzymes with such properties, AvaII is a Type II restriction endonuclease predicted to belong to the PD-(D/E)XK superfamily (10). The enzyme is specific for DNA with the G↓GWCC sequence (where W stands for an A or T base of a ‘weak’ base pair) and cleaves it to products with three nucleotide long 5′-overhangs (11). This is highly unusual for PD-(D/E)XK restriction endonucleases. In fact, only two structurally characterized enzymes, EcoO109I and BbvCI, cleave DNA with this stagger (12–14).

Structural information on AvaII was unavailable. The enzyme displays weak sequence similarity to the better characterized EcoO109I endonuclease with similar target sequence (RG↓GNCCY) (12,13). In the structure of EcoO109I in complex with dsDNA, the long α-helices of the protomers insert into the minor groove of DNA. Instead of responding to the α-helix insertion by a kink, which is typically observed e.g. in the HNH endonucleases (15), the DNA in the EcoO109I complex appears to get ‘stretched’. This feature and the scarcity of direct contacts of the enzyme with DNA bases suggest that EcoO109I, and by implication, AvaII, may have evolved from a palindrome cutter that cleaves DNA with four nucleotide stagger.

Here, we report biochemical and crystallographic experiments undertaken to better understand the unusual activity of AvaII restriction endonuclease against RNA/DNA heteroduplexes. We describe confirmation of this activity in our hands and biochemical data on the metal cation dependence of the enzyme that support its classification as a PD-(D/E)XK restriction endonuclease. AvaII could be crystallized without bound substrate, as well as in the specific complex with partially cleaved dsDNA and in the scanning complex with RNA/DNA heteroduplex. Our data suggest that AvaII can cleave RNA/DNA heteroduplexes, because it already binds dsDNA in A-like conformation. Bioinformatic analysis further suggests that this explanation may apply not only to AvaII, but to a number of other DNA heteroduplex cleaving restriction endonucleases. We have verified this hypothesis biochemically and shown that binding of dsDNA in A-like form is a good predictor of RNA/DNA heteroduplex cleavage activity.

## MATERIALS AND METHODS

### Cloning

The *R.AvaII* and *M.AvaII* genes were amplified from *A. variabilis* cells using the polymerase chain reaction and placed into the compatible high copy vector pET28a (using XhoI and NcoI restriction sites) and the low copy pACYC184 vector, respectively. The sequence encoded by the *R.AvaII* gene differed from the one present in the UniProt database (ID: Q8YYB7) by the N-terminal MGS cloning artefact and the C-terminal LEHHHHHH tag.

### Site directed mutagenesis

Polymerase chain reaction (PCR) was used to amplify *R.AvaII* gene from *pET28a_avaII* construct using primers encoding amino acid mutations of interest. PCR products were visualized using agarose gel electrophoresis. One microliter of the DpnI restriction enzyme was added to the PCR products and incubated for 1 h in 37°C. One microliter of the reaction was transformed into chemically competent *Escherichia coli* cells with *M.AvaII* gene. Selected colonies were grown for plasmid isolation in LB medium with appropriate antibiotics in 37°C overnight. The presence of the mutation was confirmed by Sanger sequencing in Genomed S.A. (Poland).

### Expression

Expression experiments were performed in the *E. coli* ER2566 strain containing the low copy vector pACYC184 with *M.AvaII* methyltransferase gene that protects bacteria against AvaII endonuclease activity. Bacterial cells were grown in the Terrific Broth (TB) with kanamycin and chloramphenicol at 37°C to an apparent optical density of 0.7 at 600 nm wavelength. Subsequently isopropyl-β-D-1-thiogalactopyranoside (IPTG) was added to the 1 mM final concentration to induce protein expression. After 18 h of expression at 22°C, the cells were collected by centrifugation.

### Purification

The bacterial pellet was resuspended in the sonication buffer (0.4 M NaCl, 50 mM Tris–HCl pH 7.6) with addition of 1 mM phenylmethylsulfonyl fluoride (PMSF). Cells were sonicated and centrifuged at 4°C and 40 000 rpm for 40 min. The supernatant was added to the equilibrated column containing Nickel-nitrilotriacetic acid (Ni-NTA) agarose resin (Qiagen). Protein was eluted with the buffer containing 0.2 M NaCl, 25 mM Tris–HCl pH 7.4, 7 mM β-mercaptoethanol and 0.1 M imidazole. AvaII was further purified by size exclusion chromatography using the 16/60 Superdex 75 column in the following buffer: 50 mM Tris–HCl pH 7.4, 0.2 M NaCl, 1 mM dithiothreitol (DTT), 5% glycerol.

### AvaII activity assay

Endonuclease activity of AvaII was assayed with pGEX-6P-3 plasmid or dsDNA and RNA/DNA heteroduplex oligonucleotides. Forty-one nucleotide long oligonucleotide sequences used for the experiments presented in Supplementary Figures S1–3 followed Murray *et al.* (8) and were provided by Purimex (Germany). The dsDNA homo- and DNA/RNA heteroduplexes were made by annealing 3′-Cy5-labeled oligonucleotides, with **GGACC** in the strand that was either DNA or RNA and **GGTCC** in the complementary DNA strand. Oligonucleotides for other AvaII assays were 3′-Cy5 labeled only in the top strand and synthesized in 0.02 μmol scale by Genomed S.A. (Poland). All substrate sequences are summarized in Supplementary Table S1. Digestion reactions were performed at 37°C for 30 min. Reaction buffer was based on the NEBuffer 4 (20 mM Tris-acetate, pH 7.9, 50 mM potassium acetate, 10 mM magnesium acetate) in which commercially available restriction enzyme AvaII (NEB) has 100% activity. AvaII was used in the amounts of 100, 10, 1, 0.1 or 0.01 pmol of dimer in 1 μl volume. Results were visualized by native polyacrylamide or agarose gel electrophoresis.

### Activity assay of enzymes predicted to cleave RNA/DNA hybrids

The RNA strand of RNA/DNA heteroduplexes was *invitro* transcribed from annealed DNA duplexes using T7 RNA polymerase prepared locally. The RNA was gel purified, dephosphorylated using FastAP (Thermo Scientific™), silica column purified (AAbiotechnology), ^32^P radiolabeled (T4 PNK Thermo Scientific™) and subsequently again silica column purified. The DNA strand of the heteroduplexes was ordered as a synthetic oligonucleotide, gel purified and radiolabeled (T4 PNK Thermo Scientific™). Next, hybrid DNA/RNA and dsDNA duplexes were prepared by mixing complementary strands with slight excess of the non-radioactively labeled strand (1.05:1.00) and annealed in the thermocycler (from 90 to 20°C within 3 h). Digestion reactions were set up in 12 μl volume using 96 fmoles of duplex per reaction, with SUPERase•In™ RNase Inhibitor 1 U/μl, in the buffer recommended by the manufacturer. Enzymes were used at a maximum concentration compatible with glycerol content lower than 4.75%. MvaI, HindIII, PvuII (Fastdigest™ series) and BcnI enzymes were from Thermo Scientific™. AvaII, HinP1I and EcoRV were from New England Biolabs.

Reactions were incubated in the thermocycler for 16 h in 37°C than stopped by addition of 14 μl of loading dye (95% formamide, 25 mM ethylenediaminetetraacetic acid (EDTA)). Standard denaturing polyacrylamide gel electrophoresis in 8 M urea was carried out. Before loading on the gel, samples were incubated at 80°C for 10 min. Cleavage products in RNA/DNA digestion reactions had to stem from the RNA/DNA heteroduplexes and not from contaminating dsDNA template, because the template was 20 bp longer than the heteroduplex. Moreover, only one radiolabeled fragment was observed, whereas two would be expected if the cleavage product was derived from transcription template dsDNA.

### Electrophoretic mobility shift assay

The gel shift assay was done with 10 pmol of Cy5 3′ labeled dsDNA and DNA/RNA heteroduplex as a substrate and 10, 20, 50 or 100 pmol of AvaII dimer in 1 μl volume. The binding reaction was performed at 37°C for 30 min in the presence of bovine serum albumin (BSA) (1 mg/ml) and reaction buffer based on the NEBuffer 4 1× (20 mM Tris-acetate pH 7.9, 50 mM potassium acetate, 10 mM magnesium acetate, H_2_O) with 10 mM calcium chloride instead of magnesium acetate. Native gel electrophoresis was run in an 8% polyacrylamide gel in 89 mM TBE or TB buffer supplemented with 5 mM CaCl_2_. After gel pre-run (10 V, 1 h) samples were loaded with 10 × loading dye solution (3 g Ficoll 400, Orange G dye, ddH_2_O up to 10 ml) and run at 90 V. Fluorescently labeled oligonucleotides were visualized using ImageQuant LAS 4000 or ChemiDoc XRS+ (Bio-Rad). Protein was stained with Coomassie Brilliant Blue solution and destained with water.

### Electrophoretic mobility shift competition assay

The gel shift assay was done with 10 pmol of 3′ Cy5 labeled dsDNA or RNA/DNA heteroduplex. 0, 5, 10, 25, 50, 100 or 200 pmol of unlabeled competitor and 10, 50 or 75 of AvaII dimer in 1 μl volume were used. The binding reaction was performed at 37°C for 30 min in the presence of BSA (1 mg/ml) and reaction buffer containing 20 mM Tris-acetate pH 7.9, 50 mM potassium acetate and 10 mM calcium chloride. AvaII protein was added last. Results were visualized by native polyacrylamide gel electrophoresis supplemented with Ca^2+^.

### Crystallization

For crystallization trials, AvaII was rebuffered into the Ca^2+^ ion containing buffer (50 mM Tris–HCl pH 7.4, 0.2 M NaCl, 5 mM CaCl_2_, 1 mM DTT, 5% glycerol). The 14 mg/ml enzyme concentration was used for all attempts except for the co-crystallization with RNA/DNA hybrid, where 24 mg/ml protein solution was applied. For the crystallization of the AvaII complexes, the protein was mixed with oligonucleotides (suspended in _dd_H_2_O) in 1:1.1 molar ratio (protein dimer to oligoduplexes). The mixtures were left on ice for 1 h and subsequently put on crystallization plates. CrystalQuick™ RW crystallization plates (Greiner Bio-One) were set up using a Phoenix (Art Robbins) or Mosquito (SPT Labtech) crystallographic robots. Crystals were grown in sitting drops at 18°C.

Crystals of AvaII alone were harvested after 2 weeks from 2 μl: 2 μl drop equilibrated against 0.5 ml of reservoir solution containing 0.03 M CaCl_2_, 0.1 M MES/imidazole, pH 6.5, 12.5% v/v MPD, 12.5% w/v PEG 1000, 12.5% w/v PEG 3350 (Morpheus screen A4 condition with MgCl_2_ eliminated).

Crystals of the AvaII in complex with partially cleaved dsDNA were collected 9 months after the setup of trials from 0.2 μl : 0.2 μl drop equilibrated against 40 μl of buffer containing 0.02 M L-Na-glutamate, 0.02 M alanine (racemic), 0.02 M glycine, 0.02 M lysine/HCl (racemic), 0.02 M serine (racemic), 0.1 M MES/imidazole, pH 6.5, 10% w/v PEG 20 000, 20% v/v PEG MME 550 (Morpheus H1 condition). The blunt ended dsDNA was obtained by annealing of 5′-GTA**GGACC**ATC-3′ and 5′-GAT**GGTCC**TAC-3′ oligonucleotides.

Crystals of AvaII in complex with RNA/DNA heteroduplex grew within a few days in 0.2 μl: 0.2 μl drops equilibrated against 0.1 ml of solution containing 10% w/v PEG 20 000, 20% v/v PEG MME 550, 0.02 of sodium formate, 0.02 M ammonium acetate, 0.02 M trisodium citrate, 0.02 M sodium potassium L-tartrate, 0.02 M sodium oxamate and 0.1 M MOPS/HEPES-Na, pH 7.5 (Morpheus G5 condition). The RNA/DNA heteroduplex was composed of the 5′-GUA**GGACC**AUG-3′ (RNA) and 5′-CCAT**GGTCC**TA-3′ (DNA) oligonucleotides. Glycerol was used in cases when crystals could not be safely cryo-cooled without any protection.

### Data collection and structure determination

The structure of AvaII in complex with RNA/DNA heteroduplex was solved by the SIRAS method with the help of an iodide derivative. The derivatization was done by soaking crystals for a few minutes in 0.5 M NaI solution in the reservoir buffer. The best native and derivative data for the crystals of the AvaII–RNA/DNA complex were collected at the P13 beamline of the PETRA III synchrotron ring (EMBL/DESY, Hamburg, Germany). The native data was obtained at 1.2782 Å wavelength and reached 1.8 Å resolution. The derivative data was collected at 2.0664 Å wavelength. Unless stated otherwise, the data were processed and scaled with XDS (16). The SHELXC program (17) indicated the presence of significant anomalous signal reaching about 3 Å resolution. Four heavy atom sites were found with the SHELXD program (18). The phasing with SHELXE (19) indicated a mild preference of the original hand (pseudo-free correlation coefficient (CC) of 58%, contrast of 0.58 and connectivity of 0.75) over its inverted alternative (52%, 0.46 and 0.74, respectively). The enantiomers could be clearly distinguished after SHELXE model building (CC values of 40% and 13%). The experimental phases for the correct hand were used for a few rounds of model building with the help of ARP/wARP (20) alternated with manual model improvement in COOT (21). The RNA/DNA duplex was built manually in very poor density and could only be refined with strict restraints and high temperature factors.

The data for the crystal of AvaII in the absence of any nucleic acids was collected at 0.97625 Å wavelength, at the IO3 beamline of Diamond Light Source synchrotron (DLS, Didcot, UK). The crystal diffracted to 2.3 Å resolution, had C2 symmetry and contained two protein dimers in the asymmetric unit. The data were processed with DIALS (22) and scaled with AIMLESS (23). The structure was solved by molecular replacement with the AvaII dimer and the MOLREP program (24) and rebuilt with the help of the ARP/wARP (20).

The data for AvaII in complex with partially cleaved dsDNA were collected at 0.9184 Å wavelength at the 14.1 beamline of the BESSY synchrotron (Berlin, Germany) and reached 1.9 Å resolution. The crystals had P2(1) symmetry and contained one AvaII–dsDNA complex in the asymmetric unit. The structure was solved by molecular replacement with the help of a single protomer of the enzyme by the BALBES program (25). The model refined by REFMAC program (26) within the BALBES suite was then manually striped of the high B-factor regions and submitted to the automatic model building with the Buccaneer (27) and ARP/wARP (20) programs, which built 446 out of 476 amino acids of the protein dimer. The dsDNA was then manually introduced in COOT (21).

All structures were refined with REFMAC (26) and PHENIX (28). The twin refinement was applied for the structure of AvaII alone. The analysis of the diffraction data clearly indicated pseudo-merohedral twinning. Two-fold twin axis with h,-k,-l operator and refined twin ratio of ∼70% was detected. The data collection and refinement statistics are presented in Table 1.

**Table 1.** Data collection and refinement statistics

	Free AvaII	AvaII RNA/DNA scanning complex	AvaII dsDNA partially cleaved complex
**Data collection statistics**			
Space group	*C* 2	*P* 2_1_	*P* 2_1_
Cell dimensions			
a (Å)	73.2	37.7	37.1
b (Å)	102.7	104.0	116.2
c (Å)	121.2	78.3	56.8
β (°)	90.7	93.3	102.9
Wavelength (Å)	0.97625	1.2782	0.9184
Resolution range (Å)	53–2.35	52–1.8	40–1.9
lowest shell	53–9.1	52–8.05	40–5.64
highest shell	2.43–2.35	1.85–1.80	2.01–1.90
Total reflections	122 035	140 005	139 044
Unique reflections	37 373	53 209	36 585
Completeness (%)*	99.9 (99.0, 99.8)	95.4 (86.6, 97.2)	98.5 (98.2, 97.6)
Multiplicity *	3.3 (3.1, 3.1)	2.6 (2.8, 2.6)	3.8 (3.7, 3.7)
Mean *I*/σI*	5.5 (10.9, 1.8)	13.0 (28.8, 1.9)	10.6 (35.1, 1.4)
*R*_sym_ (%)*	9.9 (6.4, 45.2) ^‡^	4.1 (3.2, 63.8)	8.1 (2.9, 80.7)
*R*_meas_ (%)*	11.9 (7.7, 55.0)	5.1 (4.0, 79.8)	9.4 (3.4, 94.3)
CC_1/2_ (%) *	98.9 (98.9, 76.4)	99.8 (99.7, 57.0)	99.8 (99.9, 61.5)
Solvent content (%)^†^	42	51	32
B(iso) from Wilson (Å^2^)	53.5	42.2	36.1
**Refinement statistics**			
Protein atoms excluding H ^$^	7364	4629	5417
Solvent molecules	97	277	487
*R*_cryst_ (%)	21.63	16.75	17.6
*R*_free_ (%)^#^	25.83	20.21	21.7
RMSD bond lengths (Å)	0.008	0.024	0.008
RMSD angles (°)	1.1	1.3	1.2
Ramachandran (%)			
favored region	97.6	99.2	98.5
allowed region	100.0	100.0	100.0
MolProbity clashscore	1	1.7	0.9
PDB accession code	6S58	6G3B	6S48

*Lowest and highest shell in brackets.

^†^Calculated for protein and DNA if applicable, without bound metal ions.

^‡^ *R*_merge_ from Aimless.

^$^Alternative conformations treated separately.

^#^5% of reflections were set aside randomly.

### Comparative sequence and structural analysis

Comparative sequence analysis and phylogenetic tree construction were based on a manual update of a previously published PD-(D/E)XK restriction endonuclease alignment (10). We selected three most conserved blocks of the alignment corresponding to the α-helix and two key β-strands holding the catalytic residues of the PD-(D/E)XK motif and added enzymes that were recently structurally characterized or were of interest due to RNA/DNA cleavage properties. Structural comparisons and structure based trees were done using the DALI server (29). Computational grafting of 2′-OH groups on 2′-deoxyriboses was done with the CNS program (30), enforcing correct chirality with a highly weighted constraint. The analysis of oligoduplexes present in the crystal structures and the generation of idealized nucleic acid models was done using the 3DNA program (31).

We have previously tested how severe steric conflicts need to be to interfere with DNA binding (32). This study for DNA base anchored methyl groups suggested that clashes of up to 1.1 Å can be compensated by adaptations of protein and nucleic acids. For the present problem, flexibility of DNA backbone (rather than of bases) and the shape of a 2′-OH group (rather than the shape of a methyl group) play a role. We accounted for the smaller size of the 2′-OH compared to a methyl group, and suspect that the threshold for accommodation of clashes is likely to be similar.

## RESULTS

### AvaII activity on dsDNA and RNA/DNA, metal dependence

**Figure 1.**
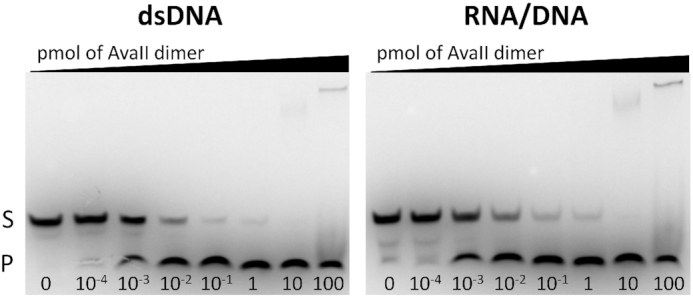
AvaII activity on dsDNA and RNA/DNA oligonucleotide heteroduplexes. A total of 10 pmoles of dsDNA or RNA/DNA were incubated for 30 min at 37°C with amounts ranging from 10^−4^ to 100 pmoles of the AvaII dimer. S: substrate; P: product.

As previously reported (8), AvaII cleaves not only dsDNA, but also RNA/DNA heteroduplexes (Figure 1). AvaII activity on both dsDNA and DNA/RNA substrates requires Mg^2+^ or Mn^2+^, but not Ca^2+^ ions, as expected for a PD-(D/E)XK enzyme (33) (Supplementary Figure S1). Activity persisted when Mg^2+^ and Ca^2+^ ions were both present (Supplementary Figure S2).

### Lower AvaII activity on RNA/DNA heteroduplexes is due to reduced affinity

The lower activity of AvaII against RNA/DNA compared to dsDNA could result either from reduced affinity or a lower catalytic rate. We carried out electrophoretic mobility shift assays (EMSAs) in the presence of Ca^2+^ ions, which support nucleic acid binding but not catalysis. For dsDNA, a stoichiometric amount of AvaII was sufficient to shift most of the DNA band. A several-fold higher amount of AvaII was required to equally deplete the free RNA/DNA heteroduplex (Supplementary Figure S3). In order to further compare affinities for dsDNA and RNA/DNA heteroduplexes, we carried out competition assays, using both cognate and non-cognate duplexes, under conditions that prevent DNA cleavage. Specific complexes were only observed with cognate dsDNA, that could not be outcompeted by a 20-fold excess of either RNA/DNA heteroduplex, or non-cognate dsDNA heteroduplex (Supplementary Figure S4). Together with the observation that large amounts of AvaII could overcome its lower activity on RNA/DNA compared to dsDNA, this finding suggests that the AvaII binding affinity for cognate RNA/DNA is much lower than for dsDNA.

### Crystals of AvaII alone, with partially cleaved dsDNA and RNA/DNA

AvaII was prepared for crystallization in the presence of 5 mM Ca^2+^ ions. Crystals of the enzyme could be grown in the absence of any nucleic acid, in the presence of dsDNA and RNA/DNA hybrid (Table 1). As the crystals of AvaII–RNA/DNA complex were available first, the phases were obtained for this crystal form by the quick halide soaking method (34). The resulting model was then used to solve the other structures by molecular replacement.

Upon crystallization, the RNA/DNA duplex remained uncut. In contrast, dsDNA was partially cleaved, due to either trace amounts of Mg^2+^ ions in the crystallization mix (resulting from reagent impurities), or the presence of Ca^2+^ ions and the long time for crystallization (9 months). The appearance of partial DNA cleavage in the electron density could be due to A- and T-strands being cleaved only in some asymmetric units of the crystal. Alternatively, symmetry breaking at the center of the recognition sequence could lead to differences in cleavage rates for the A- and T-strands, so that one strand is cleaved, and the other is not, throughout the crystal. Averaged by the two binding modes, the same density as for partial A- and T-strand cleavage would be expected for this scenario as well.

**Figure 2.**
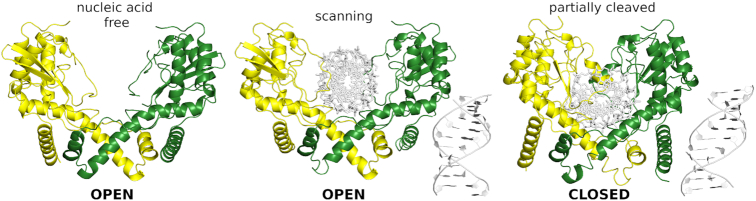
AvaII in open and closed conformations. Open form is observed for the enzyme in nucleic acid free state and in the scanning complex with RNA–DNA heteroduplex. Closed form is captured in the complex with partially cleaved dsDNA. The protein homodimer is colored yellow/green and shown in ribbon representation and the nucleic acids are depicted as white sticks.

AvaII forms symmetric or nearly symmetric dimers in all three structures. The dimerization mode is very similar, but there are considerable differences in the relative arrangement of the core regions of the two protomers. Broadly, the structures fall into two categories. An ‘open’ conformation of the AvaII dimer is observed in the absence of nucleic acids and in complex with the RNA/DNA heteroduplex. In this form, the AvaII central channel is too wide for base-specific interactions with the bound oligoduplex and the enzyme only contacts the phosphodiester backbone. Therefore, we interpret the AvaII–RNA/DNA structure as a scanning complex. Trapping of the scanning complex despite the presence of the AvaII target sequence in the RNA/DNA heteroduplex is likely the result of ∼100-fold weaker binding of AvaII to RNA/DNA than to dsDNA. In the absence of nucleic acids a second protein dimer fills in the space between the protomers that should be occupied by the DNA double helix (Supplementary Figure S5). A ‘closed’ conformation is observed in the complex of AvaII with partially cleaved dsDNA. In the closed complex, the DNA is bound to the enzyme by a set of base specific as well as backbone interactions. Moreover, the catalytic sites are optimally positioned in the close vicinity of the partially cleaved phosphoester bonds. We therefore interpret this complex as specific (Figure 2).

### AvaII overall structure

**Figure 3.**
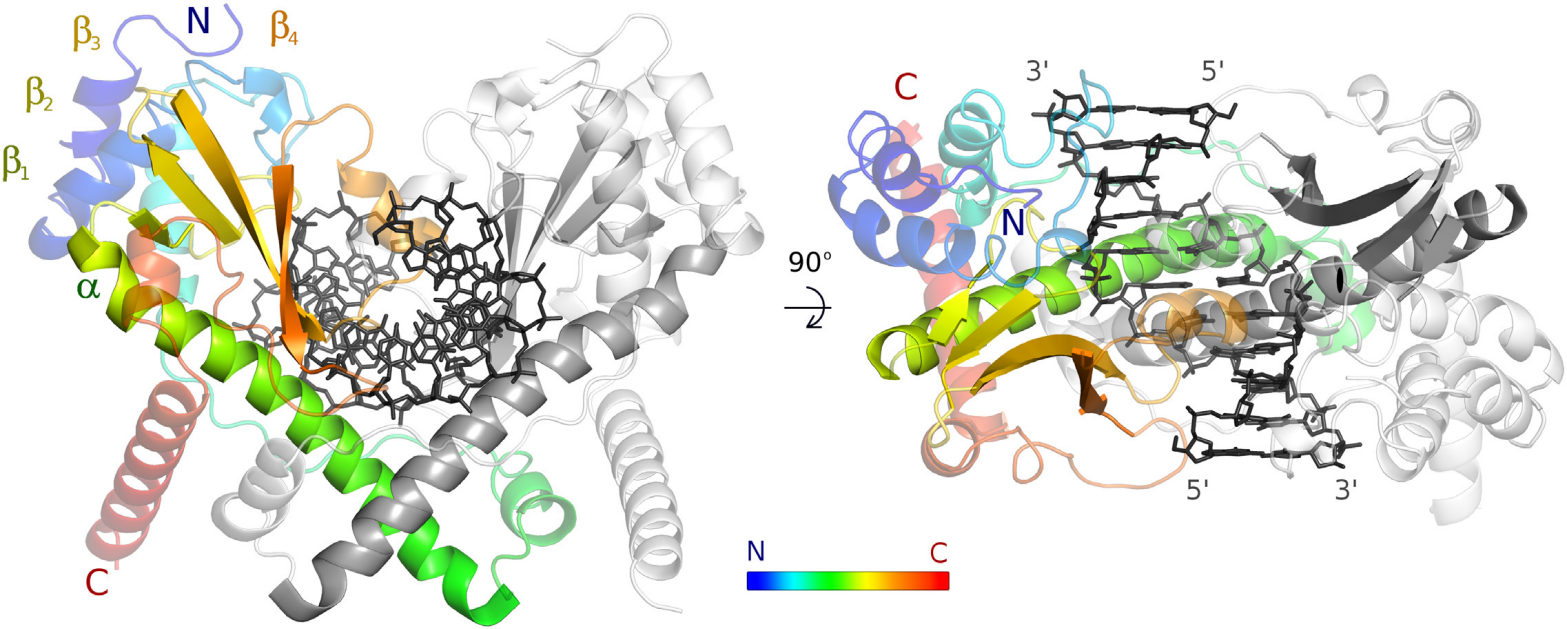
AvaII overall structure. AvaII dimer with specifically bound dsDNA seen looking onto the vertically oriented dimer axis (left), and looking down the dimer axis (right). One of the AvaII protomers is colored in rainbow representation (blue to red from N- to C-terminus), the other one is shown in gray. DNA is shown in black.

The core of the AvaII protomer adopts the same conformation in all three structures, and consists of the characteristic αββββ motif of the PD-(D/E)XK endonucleases. The helix of the core motif is over 30 amino acids long and comprises one or two short 3_10_-helices and a long α-helix (residues 98–133). Immediately downstream of the helix is the ‘canonical’ β-sheet. Its four strands comprise residues 135–138, 147–152, 156–165 and 182–188. As in other PD-(D/E)XK restriction endonucleases (35), three β-strands are antiparallel, and the fourth runs parallel to the third. The first two β-strands are connected by an extended loop, the second and third strand by a tight hairpin (Figure 3).

AvaII has both N- and C-terminal extensions to the catalytic core region. The extensions are predominantly α-helical in structure. Three most N-terminal helices (residues 9–18, 25–37 and 56–68) form a three-helix bundle that is stacked against the catalytic core (colored blue to cyan in Figure 3). The predominantly helical dimerization region is alternatingly built from fragments of the two protomers (green and red in Figure 3). Due to this highly intertwined arrangement, dimerization buries a considerable solvent-accessible surface area of about 4500 Å^2^, even though the region is fairly compact. Interestingly, the dimerization mode is very similar in all forms of AvaII. The change in the overall conformation of the dimeric enzyme is instead largely due to the straightening of the long helix of the catalytic motif in the productive complex of the enzyme (Supplementary Figure S6). From sequence analysis alone, this conformational change would not have been expected because a helix-breaking proline residue is not present in the kink region.

### AvaII active site

**Figure 4.**
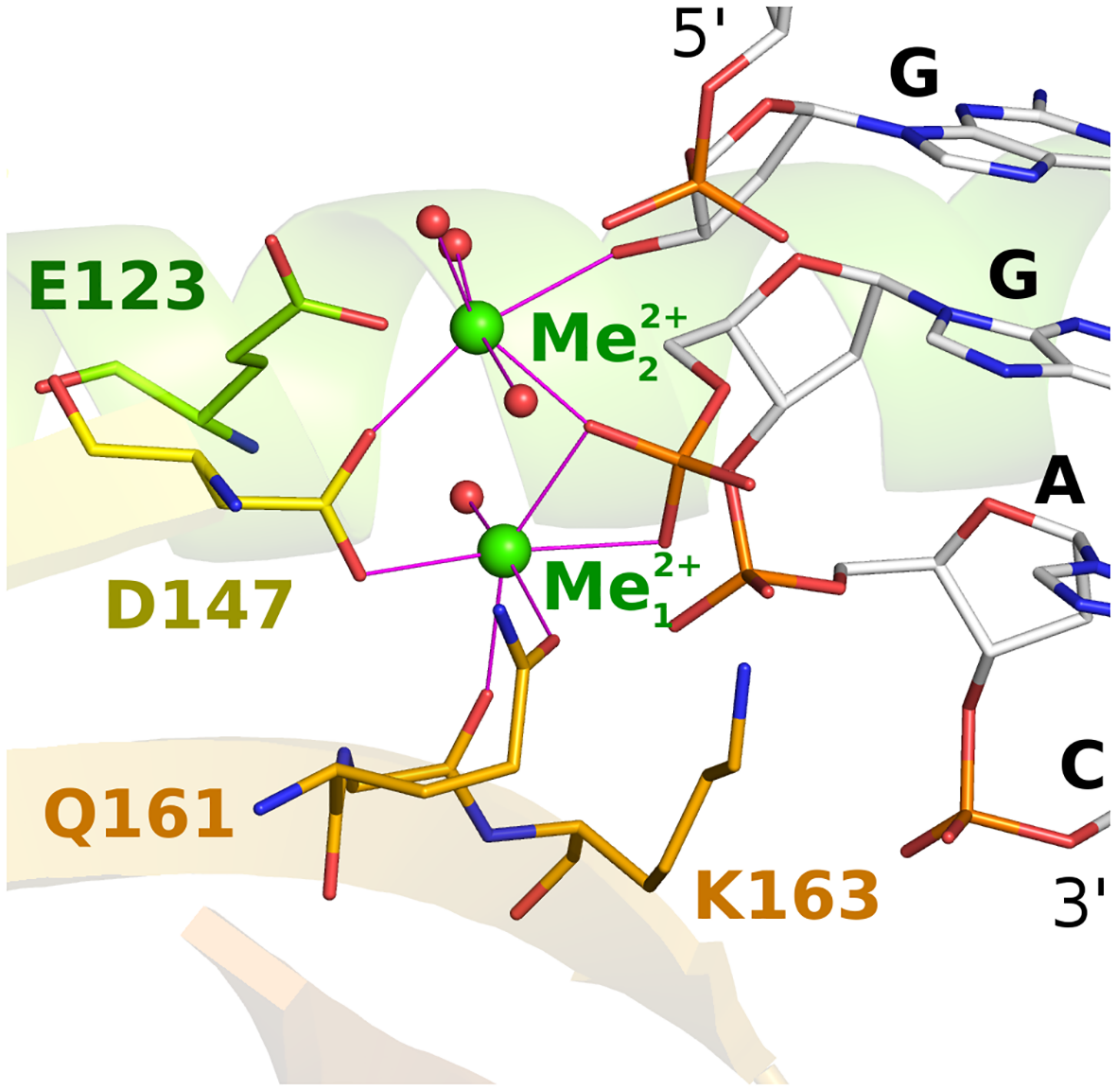
AvaII active site. Selected active site residues around the two catalytic metal ions are shown in all atom representation. DNA is shown in the region around the scissile phosphodiester bond. The figure is based on the coordinates for the complex with partially cleaved DNA, but for clarity, only the cleaved DNA conformation is shown. The color coding is as in Figure 3. Metal ions are shown in green.

AvaII exhibits a fairly canonical PD-(D/E)XK restriction endonuclease active site. The catalytic residues are arranged around two metal cations, termed here Me_1_^2+^ and Me_2_^2+^. The Me_1_^2+^, found in all active PD-(D/E)XK restriction enzymes, is involved in transition state stabilization, by contacting the OP1 non-bridging oxygen atom of the leaving group phosphate. It also aids activation of the attacking water or hydroxide ion nucleophile. Canonically, Me_1_^2+^ is held by the ‘PD’ aspartate, and ‘(D/E)XK’ aspartate or glutamate. In AvaII, these residues are D147 and a glutamine residue (Q161), instead of the usual aspartate or glutamate of the (D/E)XK motif. This residue interacts with the Me_1_^2+^ ion via the carbonyl oxygen of the terminal carboxamide group, and hence fulfills a role of the metal ligand. In the vicinity of Me_1_^2+^, there is also the ϵ-amino group of K163, the canonical K residue of the (D/E)XK motif. In the specific complex of AvaII with dsDNA, the attacking water molecule (hydroxide ion) is either partially or fully incorporated into the reaction product. The Me_2_^2+^, optional in PD-(D/E)XK endonucleases and in some cases replaced by a solvent molecule (e.g. in the EcoO109I complex), is also involved in the transition state stabilization. Moreover, this Me_2_^2+^ ion serves to acidify the leaving group and thus assists in its departure (35). It is held by the ‘PD’ aspartate, and an aspartate or glutamate of the catalytic core α-helix. In AvaII, these residues are D147 and E123, located in the expected spatial arrangement (Figure 4).

### Specific interactions of AvaII with dsDNA bases

**Figure 5.**
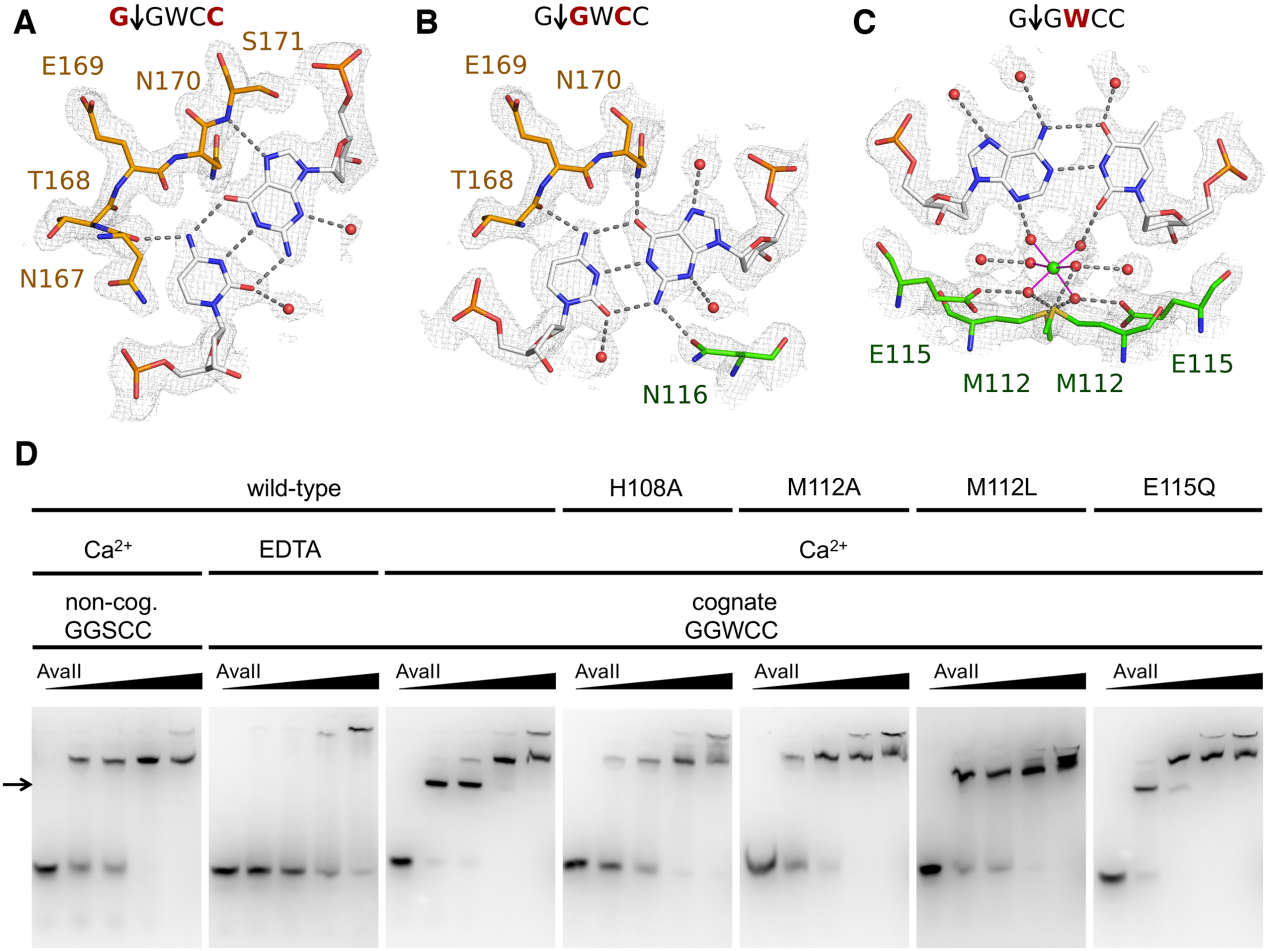
AvaII target sequence recognition. Due to the pseudo-palindromic symmetry, only a half-site of the recognition sequence is independently recognized, interactions with the other half-site are symmetric. The figure shows in the interactions of (**A**) the outer G:C pair, (**B**) the inner G:C pair and (**C**) the central A:T pair (only one of the possible A:T pair binding modes is shown for clarity). The color coding is as in Figure 4. The composite omit map was contoured at 1 rmsd. (**D**) EMSA assays for protein DNA binding with 1 μM dsDNA (29 bp), containing either a single miscognate (GGSCC) or cognate (GGWCC) site. Protein dimer amount in each series is 0, 10, 20, 50 and 100 pmol.

In the AvaII complex with partially cleaved dsDNA, the entire oligoduplex could be traced clearly. As the AvaII dimer interacts nearly symmetrically with its pseudosymmetric G↓GWCC target, it suffices to describe one half-site only (Figure 5).

The outer G:C pair is in part selected by shape recognition. Steric conflicts with the AvaII main chain of N170 and/or S171 preclude binding of pyrimidine bases in the position of the guanine. In contrast, the S171 NH group donates a hydrogen bond to the N7 atom of the purine base in this position. The choice between the purines appears to be determined by interactions with the base-paired pyrimidine. Cytosine base in this position donates a hydrogen bond to the main chain carbonyl of N167 of AvaII (Figure 5A).

The inner G:C base pair appears to be primarily selected by favorable hydrogen bonds. On the minor grove side, the exocyclic guanine amino group donates a hydrogen bond to the N116 residue of AvaII. This interaction selects for R upstream of the central W in the recognition sequence, but it does not suffice to specify the G:C base pair. Non-degenerate specificity results from additional major groove interactions. The hydrogen bond from the N170 amide nitrogen atom selects for the presence of the guanine O6 atom. A potential hydrogen bond can also be formed between the main chain carbonyl atom of T168 and the N4 atom of the cytosine, but it does not have optimal geometry (Figure 5B).

Interactions of AvaII with the central A:T pair of the recognition sequence were expected to occur in the minor groove, where hydrogen bonding patterns are helpful to distinguish W from S pairs. To our surprise, instead of an amino acid in this region, we found density for a central octahedrally coordinated atom with six solvent molecule ligands. Ligand locations and distances of around 2.2 Å suggested that the ion should be interpreted as Ca^2+^. In physiological conditions, this site may be occupied by a Mg^2+^ or Na^+^ ion of very similar coordination spheres. While the metal ion is coordinated only by solvent molecules, these in turn are anchored by hydrogen bonding interactions to the Sδ atoms of M112 and the carboxylate groups of E115, which are held by in place by contacts to H108. Irrespective of the physiological identity of the metal ion, it is likely responsible for the preference for W over S in the central position. The presence of a G:C or C:G pair would place an amino group in the central minor groove, and one of its hydrogen atoms uncomfortably close to the metal ion. To our knowledge, readout of DNA sequence by non-catalytic metal ions is novel for restriction endonucleases, and perhaps more generally for enzymes that interact with DNA (Figure 5C).

### Biochemical verification of the AvaII dsDNA interactions

To test the role of residues involved in creating the environment for the metal ion, we carried out EMSA experiments with wild-type AvaII and its variants, with cognate and non-cognate DNA, in the presence of either Ca^2+^ or EDTA (Figure 5D). First, the behavior of the wild-type enzyme was tested. A faster-migrating band, most likely corresponding to the specific complex, was observed at low AvaII concentration, with cognate DNA and in the presence of Ca^2+^ ions (marked by an arrow in Figure 5D). The disappearance of this band in favor of slower migrating band at high AvaII concentrations is not fully understood, but may result from a super-shift effect. With the non-cognate sequence and Ca^2+^, or with cognate sequence and EDTA, only the slower migrating band that we interpret as non-specific complex and the band corresponding to the DNA stuck in the loading well were observed.

Next, AvaII variants were tested. All of them migrated like the wild-type enzyme in the gel filtration, which confirms their proper folding (Supplementary Figure S7). EMSA experiments were first carried out with the cognate GGWCC substrate and Ca^2+^ ions. Specific complex was only observed for the wild-type enzyme and the E115Q variant. We conclude that the anchoring of the Ca^2+^ ion, that may be retained after the conservative substitution, is important for the specific complex formation (Figure 5D). Next, gel shift assays were performed for non-cognate sequences with alterations to the central (GGSCC) or flanking base pairs (GAACC or GATCG). The specific complex was not formed for any of the mutants and non-cognate sequences except for trace amounts observed for the variants containing the E115Q substitution (Supplementary Figure S8). Comparisons of the wild-type AvaII and its variants in the presence of EDTA were not informative, because the specific complex did not form even with the wild-type enzyme (Supplementary Figure S9). This is not surprising, since the EDTA chelates out not only the metal ion close to the central base pair, but also the active site metals, which are known to make a major contribution to DNA affinity of PD-(D/E)XK restriction endonucleases.

The importance of the M112 and E115 residues could also be shown by activity assays. The M112L and E115A substitutions severely impaired the activity compared to the wild-type enzyme. Double (M112L/E115Q) and triple (H108A/M112L/E115Q) AvaII variants had properties as expected from the behavior of single mutants. None of the mutants displayed any activity with respect to RNA/DNA hybrids (Supplementary Figures S10 and 11A). The alterations of the AvaII target sequence abolished the activity of wild type AvaII as well as its variants (Supplementary Figure S11B).

### The dsDNA in the AvaII complex has the A-form

In the AvaII–dsDNA complex, the plane of the specifically recognized base pairs is noticeably displaced from the orientation perpendicular to the duplex axis. This feature is not seen in B-DNA, but is characteristic for A-like nucleic acids (dsRNA or A-DNA). In order to make this observation more quantitative, parameters of AvaII bound dsDNA were determined using the 3DNA program (31). All four steps of the recognition sequence are classified as A-like. Displacement from the center of the helix axis is 7–8 Å for the base pairs of the recognition sequence, very close to the value expected for A-DNA (6.9 Å) but not B-DNA (1.9 Å). The minor groove of the AvaII bound dsDNA is ∼15 Å deep, and thus shallower than in ideal A-DNA (18.5 Å) and deeper than in ideal B-DNA (11.7 Å). The major groove of DNA in the AvaII complex is about 20 Å deep, more than expected for either A- (15.2 Å) or B-form (17.2 Å) nucleic acid. Some of the sugar puckers have a C3′-endo conformation characteristic for A-DNA, instead of the C2′-endo form characteristic for B-DNA. In summary, we conclude that there is strong evidence that AvaII binds dsDNA in the A-like conformation (Table 2; Supplementary Table S2 and Figure S12).

**Table 2.** Selected parameters of AvaII bound dsDNA in comparison with A-DNA, B-DNA, DNA/RNA and RNA

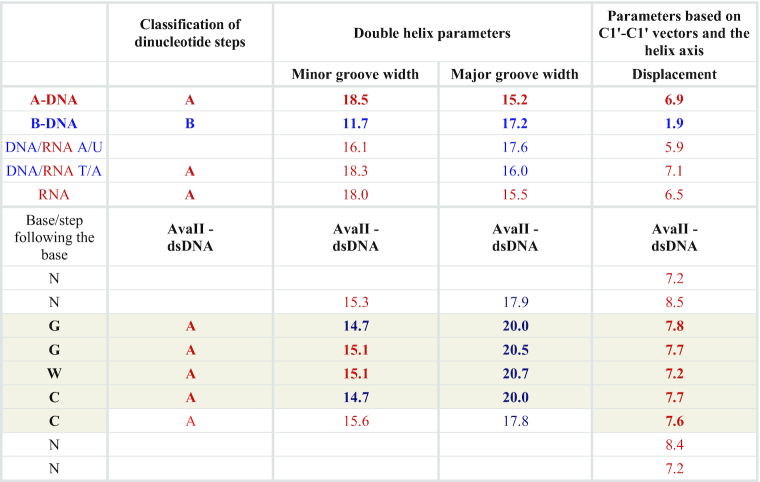

The values for two alternative conformations in the crystals were averaged. The target sequence is shaded (5 bp, 4 steps). The values similar to observed for the A- and B-like nucleic acids are marked in red and blue, respectively. Further nucleic acid parameters and comparison with EcoO109I bound dsDNA are presented in Table S1.

### Scanning complex of AvaII with RNA/DNA heteroduplex

In the crystals containing AvaII and RNA/DNA oligoduplex, the protein dimers alone form a three-dimensional lattice, with channels that extend through the entire crystal. RNA/DNA molecules bind in these channels, apparently in different registers. In these special circumstances, even complete blurring of the electron density would not be surprising. However, this does not happen and instead only discrete register shifts occur. Twelve base pairs are observed in the crystal instead of 11 chemically present in the duplex. For only two registers, the base pairs at the end of duplexes would have to be much weaker than the others. As this is not observed, we suspect that more than two different registers occur in the crystals. Moreover, RNA and DNA strands are also likely averaged. Due to the disorder of the nucleic acid component in the crystals, electron density is well defined and B-factors are in the expected range only for the protein. Nevertheless, the density is of sufficient quality to conclude that AvaII interacts only with the phosphodiester backbone of the RNA/DNA heteroduplex and does not engage in base specific contacts. Therefore, the arrangement resembles a non-specific scanning complex, or may be a crystallization artifact (Supplementary Figures S13–15).

### A model of the AvaII–RNA/DNA specific complex

**Figure 6.**
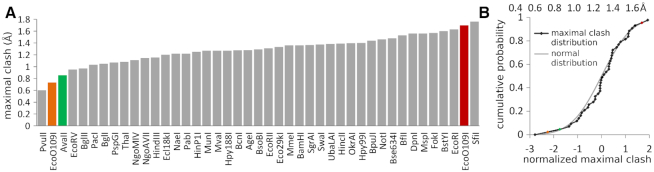
RNA/DNA binding propensities deduced from available restriction endonuclease dsDNA complex structures. (**A**) Maximal clash score values for structurally characterized Type II restriction endonucleases. For each grafted 2′-OH group, only the maximum clash was taken into account. (**B**) Cumulative distribution of maximal clashes of 2′-OH groups in Type II restriction endonucleases. The Z-scores were calculated by subtracting the average and dividing by the standard deviation. The Z-scores for EcoO109I were calculated taking into account (brown) or ignoring (orange) the outermost nucleotide pairs of the recognition sequence. The probability for a score to be this low (one sided test) or as far from the average (two sided test) is below 5% in both cases.

As we could not obtain an experimental structure of a specific AvaII complex with RNA/DNA heteroduplex, we modeled it based on the structure of the enzyme with dsDNA. As AvaII binds DNA in the A-like form, it should be able to bind RNA/DNA in the same conformation, without serious steric conflicts. In order to test this prediction, we used the CNS program (30) to computationally graft 2′-OH groups onto the AvaII bound dsDNA, one at a time, without altering 2′-deoxyribose puckers. We then assessed the extent of steric conflicts, with the AvaII dimer and with the neighboring 2′-deoxynucleotides. The results show that expected steric conflicts of 2′-OH groups in the region of the recognition sequence are very mild, and should be easily resolvable by slight structural adaptations of the enzyme and nucleic acid. We have next performed the same modeling of the 2′-OH group positions for all other restriction endonuclease-dsDNA specific complexes present in the PDB. The comparison shows that the clash score is smaller for AvaII than for almost all other restriction endonucleases (*P* < 0.05) (Figure 6).

### Predictions of RNA/DNA heteroduplexes cleaving enzymes

**Figure 7.**
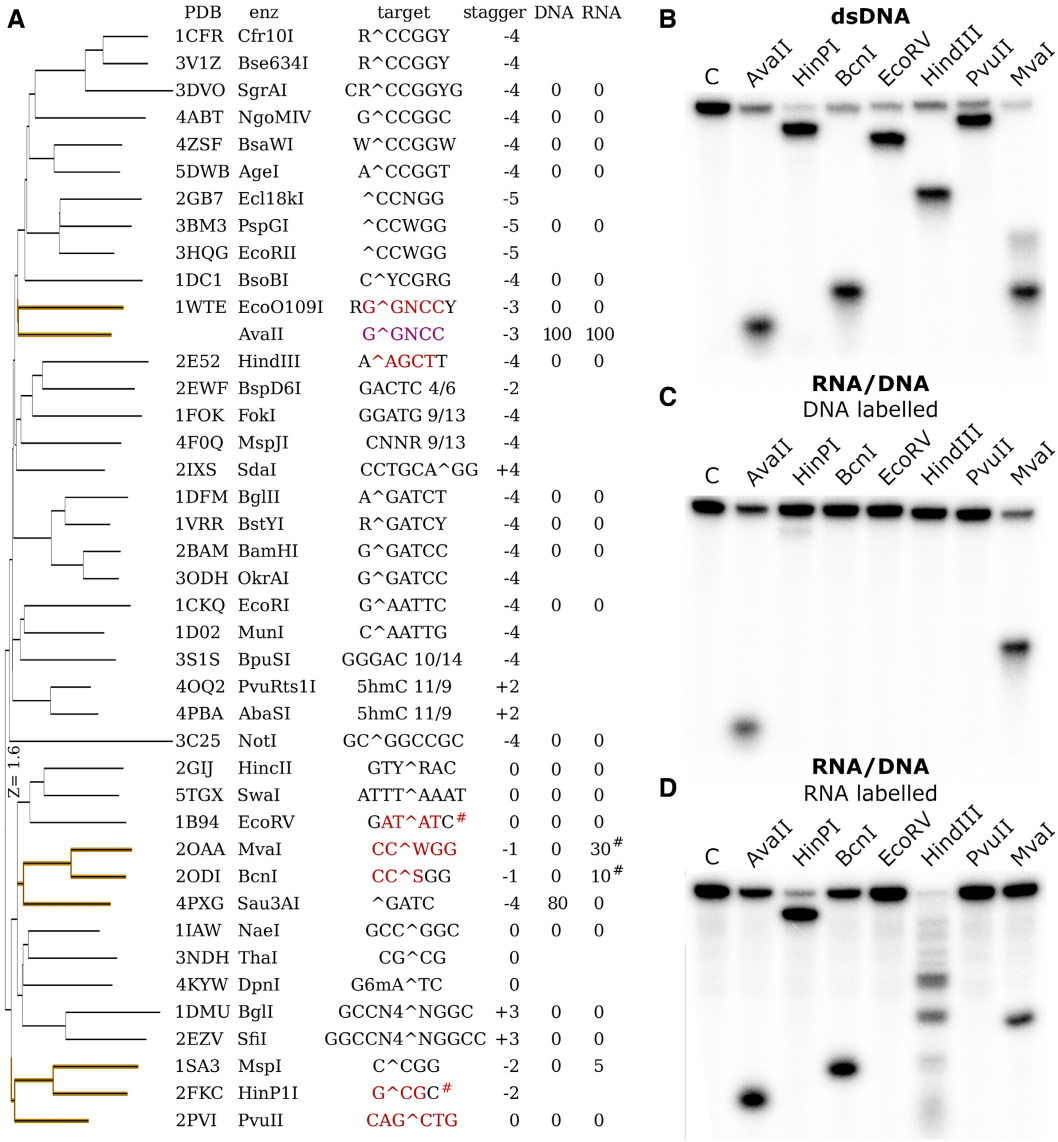
Structure based search for endonucleases that bind dsDNA in the A-like form and verification of their RNA/DNA cleaving activity. (**A**) DALI server (29) clustering of PD-(D/E)XK restriction endonucleases based on their structure. Recognition sequences are indicated, with bases bound in A-form according to the 3DNA program (31) in red. The percent cleavage of RNA and DNA strands is literature based (7). ^#^For EcoRV and HinP1I the assignment was inconsistent between the structures. (**B**) Cleavage of dsDNA with a single target site for the indicated restriction endonucleases. The top DNA strand was labeled. The digestion pattern is as expected based on the location of cleavage sites. (**C**) Cleavage of RNA/DNA, the labeled top strand is DNA. (**D**) Cleavage of RNA/DNA, the labeled top strand is RNA. The sequence of substrates was in all cases the same except for T/U differences (Supplementary Figure S15).

AvaII is not the only enzyme of known structure that binds dsDNA in A-like conformation. Among structurally characterized PD-(D/E)XK restriction endonucleases, EcoO109I, HindIII, EcoRV, MvaI, BcnI, HinP1I and PvuII have notable portions of their target DNA sequences bound in the A-like form (Figure 7A). With the exception EcoO109I, which was found not to cleave RNA/DNA heteroduplexes in the earlier screen, the enzymes are commercially available. We tested all of them on synthetic dsDNA and RNA/DNA duplexes, with one 5′ labeled strand (Supplementary Figure S16). Digestions of dsDNA illustrate the migratory behavior of reaction products (Figure 7B). For RNA/DNA substrates and standard enzyme concentrations, neither RNA nor DNA strand cleavage was observed. However, when we used the enzymes at maximum concentration (compatible with <5% glycerol in the final reaction mix), cleavage could be detected in many cases. The control AvaII and MvaI were able to cleave the DNA strand of RNA/DNA heteroduplexes (Figure 7C). Of the tested enzymes, only PvuII and EcoRV did not cleave RNA/DNA heteroduplexes (Figure 7D).

## DISCUSSION

### Specific and non-specific dsDNA complexes of restriction endonucleases

Crystals of restriction endonucleases with DNA typically trap-specific complexes. However, there are exceptions to the rule, even with the cognate substrates. In a recent crystallization study of AgeI with DNA, both specific and pre-specific complexes were trapped (36). A more extreme case is the structure of BsaWI with DNA, which surprisingly does not show any sequence specific contacts and has therefore been interpreted as an unspecific complex (37). More typically, scanning complexes have been obtained with mis- or non-cognate DNA substrates. Structures of specific and scanning complexes are available for BamHI (38,39), EcoRV (40) and BstYI (41,42). The comparison shows that the transition from non-specific to specific complexes can involve reorientation of the DNA binding mode, slight changes in the dimerization interface, conformational adjustments of the protomers and folding of the regions that interact with DNA.

Our series of structures of AvaII alone, in the scanning complex with an RNA/DNA heteroduplex and in the specific complex with partially cleaved dsDNA, nicely illustrates all these changes. The two open forms (in the absence of nucleic acids and in the scanning complex with the RNA/DNA duplex) are almost identical and no dynamic motions are found between them according to the DynDom server (43) (Supplementary Figure S17A). The closure of the enzyme on the target, resulting in a productive complex is reflected by ∼30° rotation that brings the two active sites into the optimal position for dsDNA cleavage (Supplementary Figure S17B). Interestingly, despite the similarity between the two enzymes, AvaII and EcoO109I, close up on their target sequences in slightly different ways. According to the DynDom server (43) to achieve the switch between conformations, AvaII protomers rotate around one main axis, whereas EcoO109I has more elaborate dimerization interface and therefore uses intra-protomer axes to close-up (Supplementary Figure S17C).

### AvaII and EcoO109I bind the central four dinucleotide steps in A-conformation

**Figure 8.**
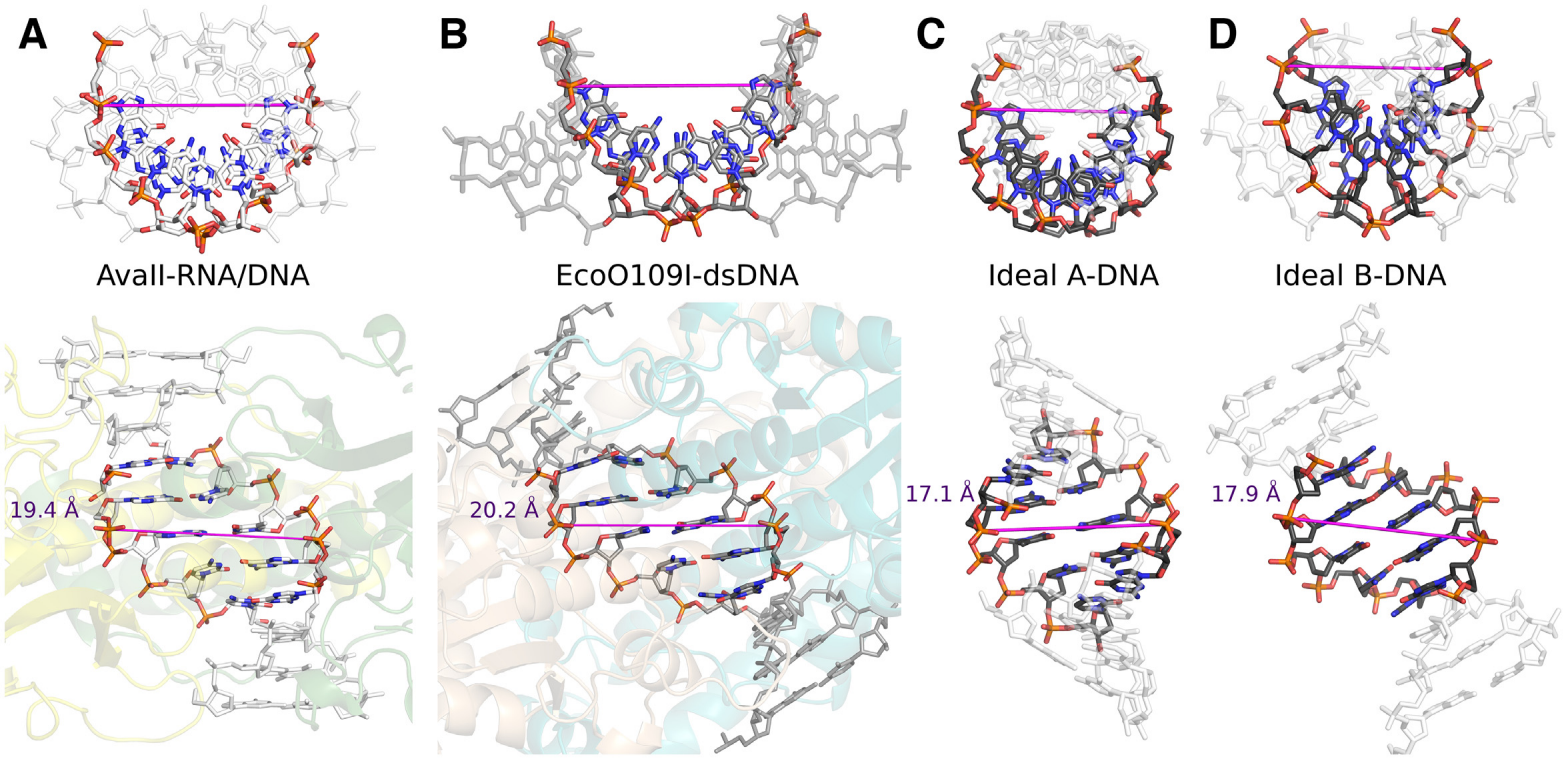
A-DNA form of AvaII bound dsDNA. Comparison of the double stranded helix conformation in (**A**) AvaII complex, (**B**) EcoO109I complex (12), (**C**) ideal A-DNA and (**D**) ideal B-DNA. Only five central base pairs of the duplexes were used for superposition. The ideal oligonucleotides were generated with 3DNA (31). The magenta line indicates the positions and distance between the phosphates of the scissile bonds in the complexes and the corresponding atoms in the duplexes.

**Figure 9.**
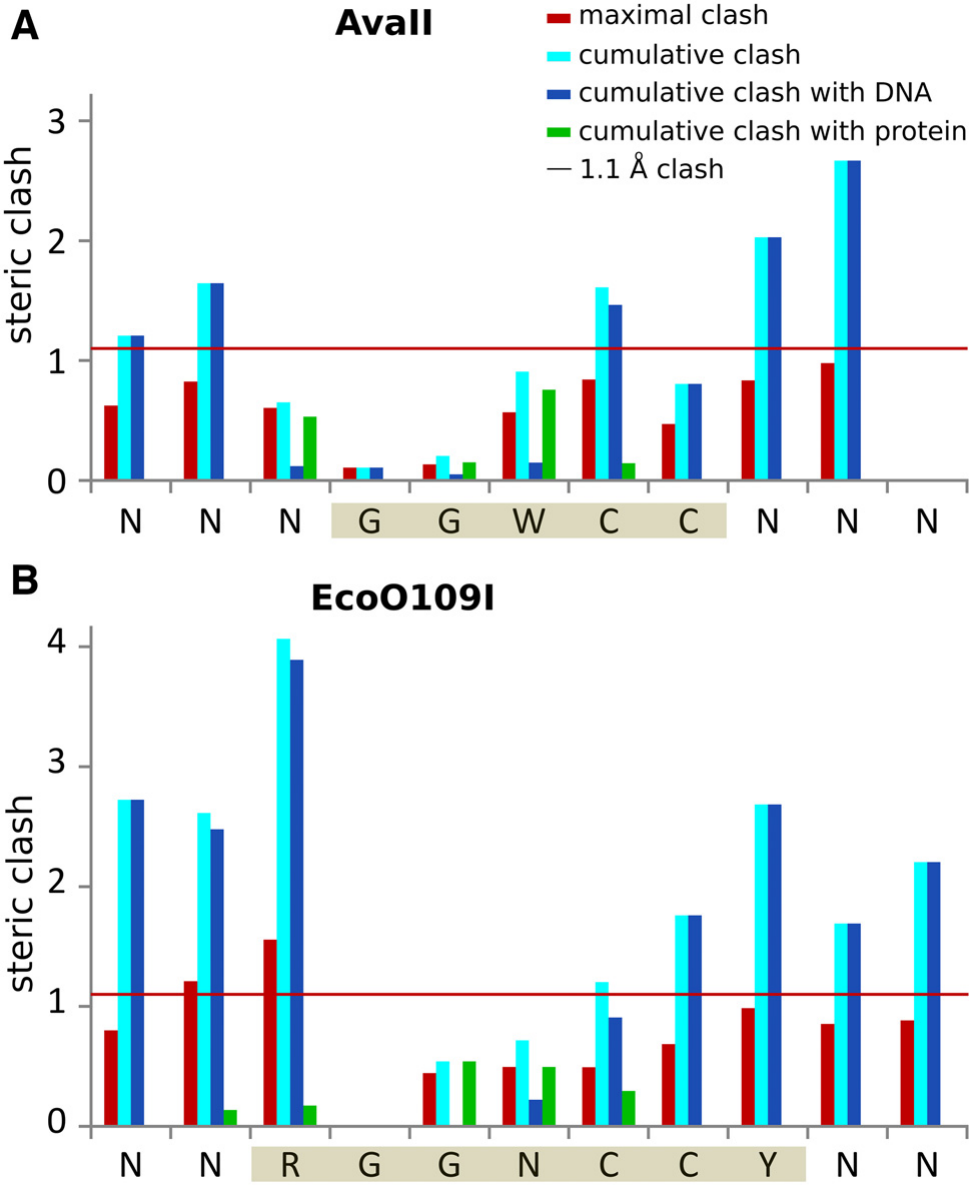
Steric conflicts of the 2′-OH groups *in silico* introduced into the AvaII and EcoO109I (13) dsDNA complexes. 2′-OH groups were grafted onto the 2′-deoxyriboses, without alteration of their puckers, with the help of the CNS program (30). Maximal and cumulative clashes were then scored as in a recent analysis of methyl group steric conflicts (32). The 1.1 Å threshold for functionally relevant steric clashes refers to methyl groups on DNA bases, but we suspect that a similar threshold applies to the clashes of 2′-OH groups (red horizontal line). For each nucleotide maximal and cumulative clash is indicated. Additionally the clashes with nucleic acid and protein were independently summed. Values were averaged for both DNA strands. The AvaII and EcoO109I target sequences are shaded in sepia.

Comparison of the structures of AvaII and EcoO109I shows that the catalytic core and its immediate vicinity are conserved, as expected. On the N-terminus AvaII has over 40 residues more than EcoO109I. On the C-terminal side EcoO109I is the more elaborate enzyme (Supplementary Figure S18). Despite these differences, the mode of dsDNA binding is similar for the two enzymes. In particular, some of the specific amino acid interactions are conserved (Supplementary Figure S19). The ‘stretched state’ of DNA found in both complexes is perhaps induced by insertion of the long α-helices into the minor groove. It finds its expression in the A-like conformation of four central dinucleotide steps of the two target sequences. Differences in DNA interactions correlate with changes in the recognition sequence. EcoO109I, which lacks the specificity for the central base pair, also lacks the metal ion that mediates this selectivity. AvaII lacks specificity for the flanking R:Y and Y:R base pairs of the EcoO109I target. The enzyme also lacks the region holding the tryptophans (W130) that in EcoO109I complex wedge into the DNA and force a transition from A-form in the center to B-form of the flanks (Figure 8 and Supplementary Figure S20). The modeling of the 2′-OH groups on the riboses of the flanking base pairs explains why EcoO109I, in contrast to AvaII, does not cleave RNA/DNA heteroduplexes (7) (Figure 9).

### RNA/DNA cleavage is licensed by the space for 2′-OH groups in A-DNA and at the interface with the enzyme

For hydrated DNA, the B-form is favored over the A-form, which is only observed in dehydrating conditions (44,45). In contrast, dsRNA adopts only the A-form, because the B-form leads to steric clashes of the 2′-OH groups. RNA/DNA heteroduplexes tend to be intermediate in structure between A- and B-forms, but closer to A-form. In some cases, the presence of a single ribonucleotide in an otherwise 2′-deoxyribonucleotide duplex is sufficient to enforce the overall A-form (46–50). Thus, the hypothesis that the endonuclease should bind dsDNA in the A-form to have the ability to cleave RNA/DNA heteroduplexes is structurally very plausible. However, the accommodation of 2′-OH groups within the nucleic acid duplex is not enough and steric conflicts with the enzyme must also be avoided.

### RNA/DNA cleaving REases share similar structures and a tendency to bind dsDNA in the A-form

**Figure 10.**
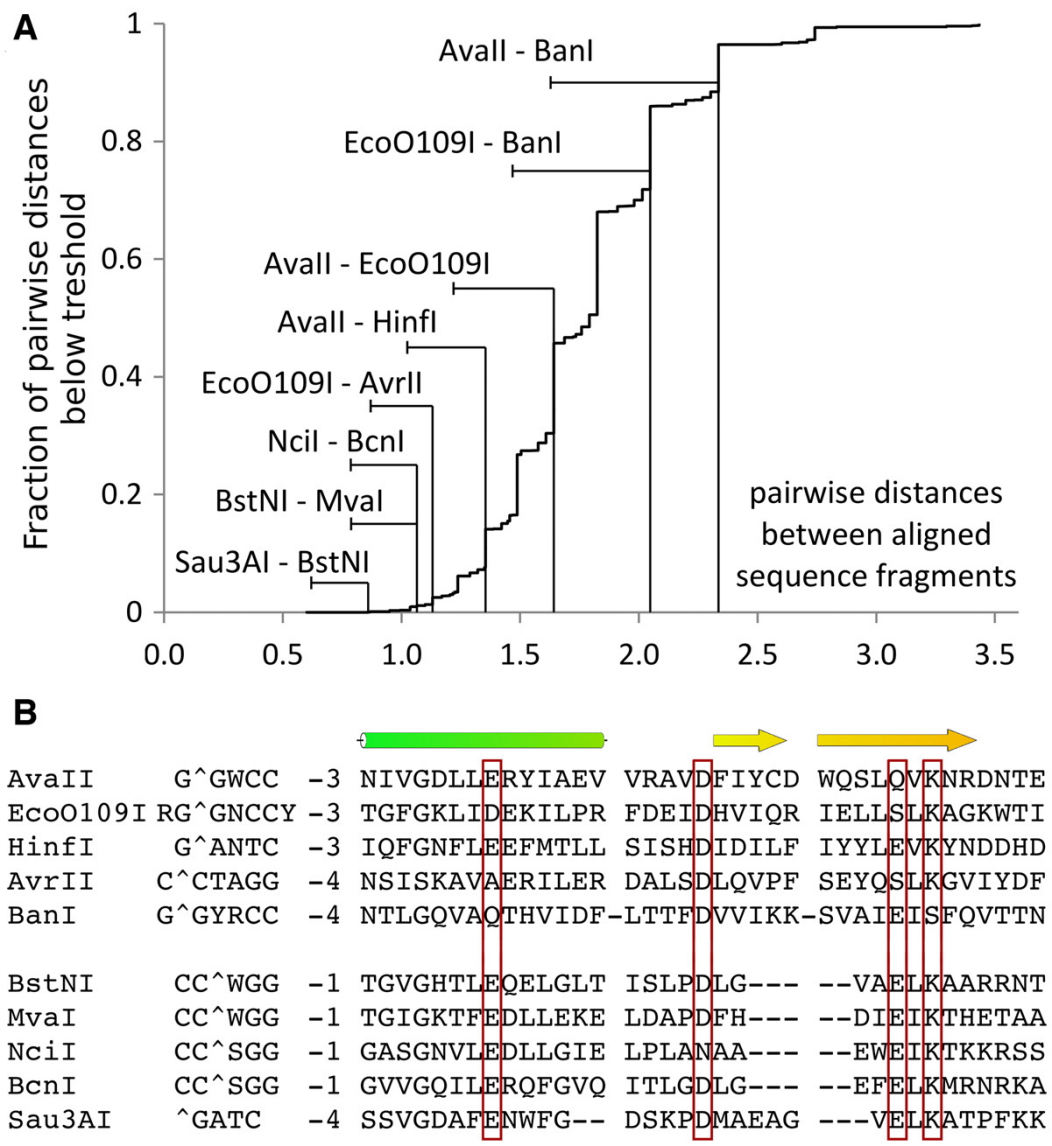
Similarity of PD-(D/E)XK restriction endonucleases that cleave RNA/DNA heteroduplexes (and of EcoO109I). (**A**) Cumulative plot of pairwise distances between the core regions of PD-(D/E)XK restriction endonucleases. Core regions were taken from a published alignment (10) supplemented with newly available data for selected RNA/DNA cleaving enzymes. (**B**) Core region alignment of two groups of heteroduplex cleaving enzymes. EcoO109I does not cleave RNA/DNA heteroduplexes, but is included in both panels for comparison.

This work has shown that restriction endonucleases that bind their target dsDNA sequences in A-like conformation are far more likely than others to cleave RNA/DNA heteroduplexes. Such enzymes tend to cluster in only a few regions of a tree of restriction endonucleases hierarchically organized according to DALI scores for structural similarity (Figure 7A). Sequence similarity in the core region of REase groups that are shown to cleave RNA/DNA heteroduplexes, is often much higher than between restriction endonucleases in general (Figure 10). Based on these observations, we suggest that there are three main groups of RNA/DNA cleaving restriction enzymes.

The first group comprises AvaII, AvrII and BanI, all previously shown to cleave at least one strand of RNA/DNA heteroduplexes (8). For both AvrII and BanI, there are hints of similarity to AvaII, but the evidence is not conclusive in either case. For AvrII, PSI-BLAST searches (51) identify EcoO109I as the most closely related restriction endonuclease in the PDB. However, the *E*-value of 1.8 is high, and the target sequence of AvrII, C↓CTAGG, does not obviously relate to the one of EcoO109I. For BanI, a similarity to AvaII is suggested by the similarity of the BanI G↓GYRCC and AvaII G↓GWCC recognition sequences and cleavage sites, which only differ by a YR replacement in BanI for the ‘stretched’ W in AvaII, Although HindIII is close to AvaII in the Figure 7, it belongs to a different clade in the DALI-tree.

The second group consists of Sau3AI, BstNI and NciI, previously shown to cleave RNA/DNA heteroduplexes (8), and MvaI and BcnI, shown to cleave RNA/DNA heteroduplexes in this work. Classification of the enzymes as a group is supported by the similarity of the structural core of Sau3AI with MvaI and BcnI (52–54) (Figure 10). Moreover, MvaI (CC↓WGG) and BcnI (CC↓SGG) cluster with BstNI (CC↓WGG) and NciI (CC↓SGG) and cleave identical recognition sequences with the same stagger.

The third group consists of MspI, previously shown to cleave RNA/DNA heteroduplexes (8) (despite insufficiently A-like structure to show up in the 3DNA classification), and HinP1I, identified as an RNA/DNA heteroduplex cleaving enzyme in this work. By similarity, PvuII should belong to this group. Its entire recognition sequence is in A-conformation (Figure 7), and the clash score for potential steric conflicts of grafted 2′-OH groups is very low. However, both the earlier experiments (7) and our tests show that PvuII does not cleave RNA/DNA heteroduplexes, and thus it does not belong to this group.

### Monomeric state and flexibility may facilitate RNA/DNA cleavage

Two groups of restriction endonucleases that bind dsDNA in A-like form and/or cleave RNA/DNA heteroduplexes consist predominantly of enzymes that either interact with dsDNA as monomers or have unknown oligomerization state. This may suggest that activity as a monomer facilitates acceptance of RNA/DNA as a substrate. Moreover, we noticed that several of the RNA/DNA cleaving endonuclease groups have considerable flexibility. The domain mobility is striking for MvaI (55) and BcnI (56), and may carry over to BstNI and NciI. The set of AvaII structures presented here also indicated substantial conformational plasticity. We suspect that the monomeric state and structural plasticity favor RNA/DNA cleavage, the former, because the concerted cleavage is not required, and the latter, because active sites are more easily repositioned to accommodate differences between dsDNA and RNA/DNA.

Despite binding dsDNA in most or all of the recognition sequence in A-form, EcoRV and PvuII do not cleave RNA/DNA heteroduplexes (Figure 7). Both enzymes are blunt end cutters. This may not be a coincidence, since the proximity of DNA cleavage sites in the two DNA strands may make it harder to accommodate the structural changes that are required to place at least one nucleic acid strand into the active site in a productive conformation. However, we also note that the REBASE (7) collection of RNA/DNA cleaving enzymes includes a blunt end cutter, PhoI, apparently identified after the original survey was published (8).

### Other folds for RNA/DNA cleavage

In restriction endonucleases, RNA/DNA cleavage is mostly catalyzed by enzymes belonging to the PD-(D/E)XK fold, but other folds dominate among enzymes that cleave RNA/DNA as their physiological substrate.

HNH endonucleases may appear as good candidates for RNA/DNA cleavage, as insertion of an α-helix into the minor groove of bound dsDNA widens the groove and should favor an A-like DNA form (15). However, at least nine HNH restriction endonucleases were tested, and only PflMI was found to cleave RNA/DNA. Among other nucleases, Cas9 is a very clear example for an HNH enzyme that can cleave RNA/DNA. It unwinds dsDNA and binds it as an RNA/DNA complex of guide RNA (gRNA) and the target DNA strand, and as a separate, displaced non-target DNA strand. The single stranded DNA cleavage is performed by RuvC domain of Cas9 whereas its HNH domain catalyzes cleavage of the DNA strand in the heteroduplex (57,58).

DDE endonucleases are another family of endonucleases that appear to be generally suitable for cleavage of RNA/DNA. Examples can be found in at least two major subgroups. Argonaute (Ago) proteins are typically eukaryotic RNA directed RNA endonucleases, and prokaryotic DNA directed DNA endonucleases (59). Nevertheless, some Argonaute proteins can also cleave the RNA strand of RNA/DNA hybrids (60). RNaseH enzymes are the physiologically most widespread group of proteins that cleave RNA/DNA, for removal of Okazaki fragment RNA primers and excision of erroneously incorporated RNA nucleotides from DNA (61).

HNH and DDE endonucleases are unrelated to PD-(D/E)XK enzymes, but share mechanistic similarities. In the case of the HNH nucleases, the analogies cover only transition state stabilization and facilitation of leaving group departure, and in the case of the DDE endonucleases, also the nucleophile activation (62).

### Precision tools for manipulating single stranded RNA

When this work was initiated, tools for precise cleavage of single stranded RNA in defined sites were missing. Since then, many possibilities have emerged, especially when the use of a ‘helper’ RNA- or DNA-strand is accepted. With an RNA helper strand, Cas13a (63), Cas13b (64), Cmr complexes (65), DNase inactivated Csm complexes (6), or Cas9 proteins with or without PAMmer (66,67) can be used for RNA-directed RNA cleavage. With a DNA helper strand, the same can be achieved with some prokaryotic Agos (60). In most cases, the cleavage site(s) are defined or at least approximately defined. RNaseH can be used to obtain sequence non-specific cleavage in the presence of a DNA helper strand. Some sequence specificity has been engineered into RNaseH with the addition of zinc fingers and mutations that suppress non-specific cleavage (68).

RNA/DNA cleaving restriction endonucleases that cut the RNA strand provide an alternative, and add a layer of protein mediated sequence specificity on top of the RNA-mediated specificity, exploited by the CRISPR related tools. Our analysis explains the finding that RNA/DNA cleaving ability is rare in Type II restriction endonucleases. We provide a structural basis of such activity for a substantial fraction of the studied enzymes. Perhaps more importantly, our analysis suggests that there is a group of enzymes that are ‘close’ to RNA/DNA directed activity from a structural biologist's perspective. We expect that these enzymes would be easier to engineer or evolve into RNA/DNA cleaving enzymes than other restriction endonucleases.

## DATA AVAILABILITY

The atomic coordinates of the refined models and the corresponding structure factors were deposited at PDB with the following accession codes: 6S58 (AvaII alone), 6S48 (AvaII-partially cleaved dsDNA) and 6G3B (AvaII–RNA/DNA scanning complex).

## Supplementary Material

gkaa403_Supplemental_File
