# Supplementary material for: Restriction endonucleases that cleave RNA/DNA heteroduplexes bind dsDNA in A-like conformation
Source: Nucleic Acids Res. 2020 May 27;48(12):6954–69. doi: 10.1093/nar/gkaa403 (PMC7337904; doi:10.1093/nar/gkaa403)
Supplement: gkaa403_Supplemental_File [file gkaa403_supplemental_file.pdf]

Supplementary Material for:

Restriction endonucleases that cleave RNA/DNA heteroduplexes bind dsDNA in A-like conformation

Marlena Kisiala[1,2,3], Monika Kowalska[1], Michal Pastor[1,2], Henryk J. Korza[4], Honorata Czapinska[1,2,$], Matthias Bochtler[1,2,$]

[1]*International Institute of Molecular and Cell Biology, Trojdena 4, 02-109 Warsaw, Poland*

[2]*Institute of Biochemistry and Biophysics PAS, Pawinskiego 5a, 02-106 Warsaw, Poland*

[3]*Biological and Chemical Research Centre, University of Warsaw, Zwirki i Wigury 101, 02-089 Warsaw, Poland*

[4]*Syngenta, Jealott's Hill International Research Centre, Bracknell, Berkshire, RG42 6EY, UK*

Corresponding authors:

Tel: +48225970732; +48225925790

e-mail: mbochtler@iimcb.gov.pl, honorata@iimcb.gov.pl

Supplementary Figures

Fig. S1

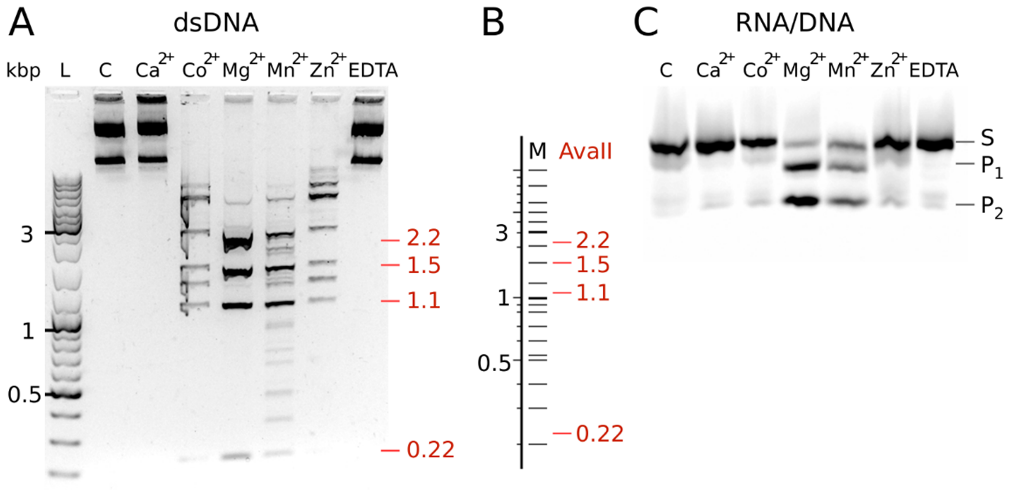

Fig. S1: Metal ion dependence of AvaII activity tested using plasmid dsDNA and RNA/DNA heteroduplexes as substrates. AvaII was incubated for 30 min at 37 ºC with its substrates and 10 mM of indicated divalent ions. **(A)** 1 pmol of AvaII and 0.05 pmol of pGEX-6P-3 were used. C - control without AvaII, L -GeneRuler DNA Ladder Mix, ThermoScientific. **(B)** Predicted products of pGEX-6P-3 plasmid digestion with AvaII generated with NEBcutter with NEB 2-log marker. Additional shorter products observed for digestion in the presence of Mn^{2+} likely result from star activity. Additional longer products are likely due to incomplete digestion. **(C)** 10 pmol of AvaII and 10 pmol of 41 nt long RNA/DNA heteroduplex were used. C - control without AvaII, S – substrate, P_1 - 30 nt product, P_2 - 14 nt product. The metal ion dependence of AvaII activity is typical of PD-(D/E)XK restriction endonucleases. The specific reaction products are marked

Fig. S2

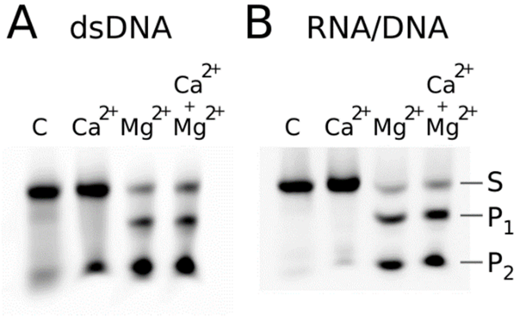

Fig. S2: Activity assay in the presence of metal ions that support (Mg^{2+}) and do not support (Ca^{2+}) activity. AvaII was incubated with dsDNA or RNA/DNA heteroduplex at 37 ºC for 30 min. **(A)** 10 pmol of dsDNA and 1 pmol of AvaII (dimer) were used. **(B)** 10 pmol of RNA/DNA heteroduplex and 10 pmol of AvaII (dimer) were used. C - control without AvaII, S – substrate, P_1 - 30 nt product, P_2 - 14 nt product. AvaII activity in the presence of Mg^{2+} ions was not blocked by the additional presence of Ca^{2+} ions.

Fig. S3

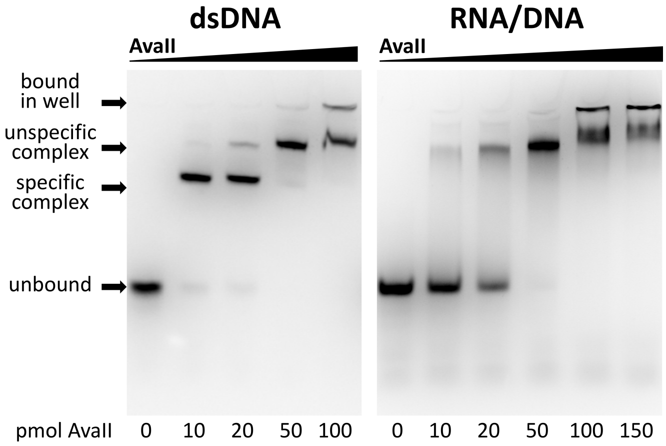

Fig. S3: Electrophoretic mobility shift assay (EMSA) to monitor complex formation between AvaII and dsDNA (left) or RNA/DNA heteroduplex (right). 10 pmol dsDNA (left) or RNA/DNA heteroduplex (right) was incubated with increasing amounts of the AvaII dimer (0, 10, 20, 50, 100 and 150 pmol) in the presence of Ca^{2+} ions that support nucleic acid binding, but not hydrolysis. In the case of dsDNA, a stoichiometric amount of AvaII suffices for an almost complete shift. In the case of the RNA/DNA heteroduplex, a five-fold excess of enzyme over substrate is needed for comparable shifting. The data indicate that AvaII has at least five-fold higher affinity for dsDNA compared to RNA/DNA heteroduplex. The band corresponding to specific AvaII – substrate complex was observed only for dsDNA. We interpret the upshifted band seen at high enzyme concentrations for dsDNA and RNA/DNA as an unspecific complex.

Fig. S4

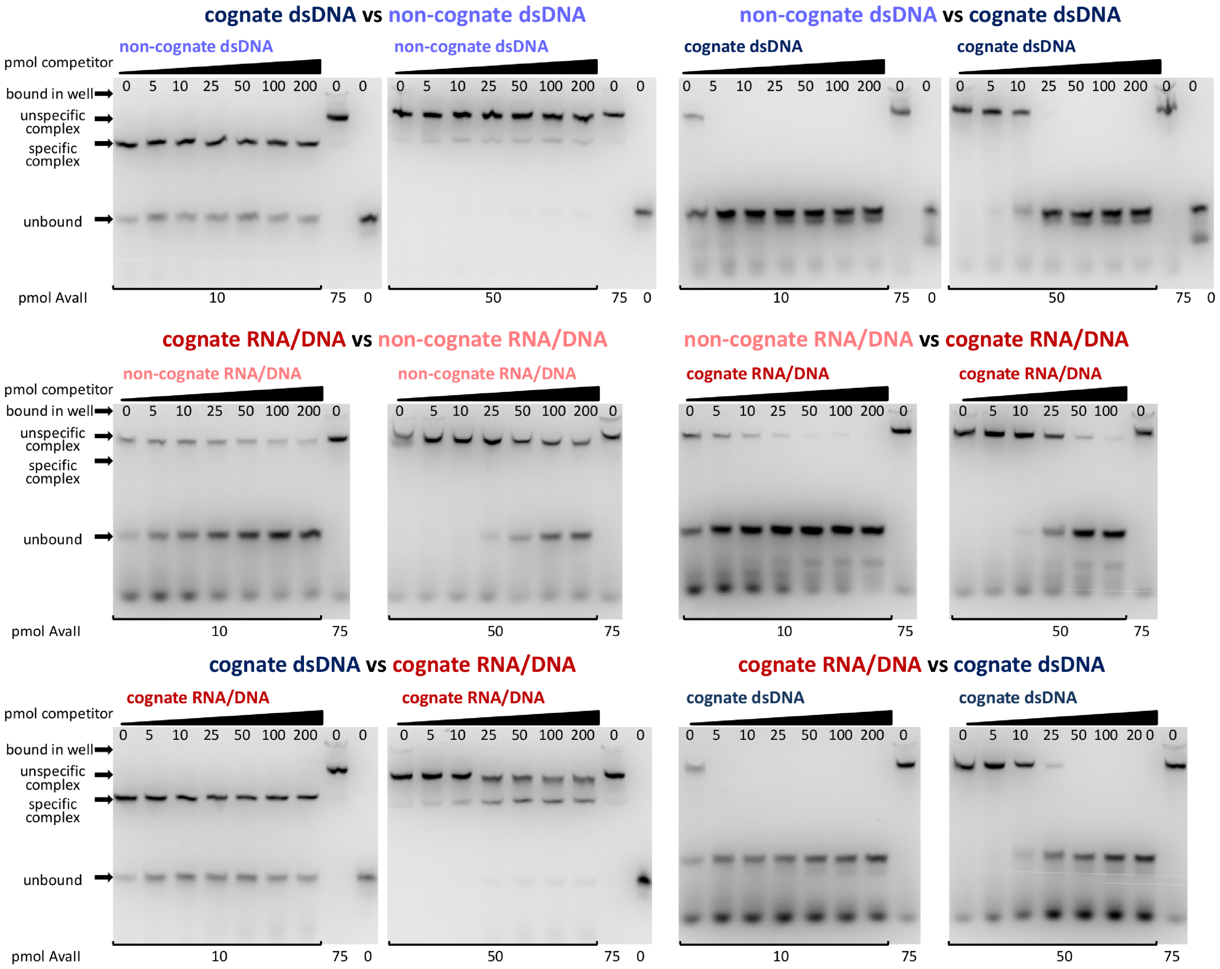

Fig. S4. AvaII competition assay. 10, 50 or 75 pmols of AvaII were mixed with 10 pmol of unlabeled oligoduplexes and titrated with increasing amount of unlabeled competitor oligoduplex (indicated above the triangular bar). The reactions were carried out in buffer containing 10 mM $CaCl_2$ for 30 min at 37 °C. The native PAGE was run in TB buffer supplemented with $CaCl_2$.

Fig. S5

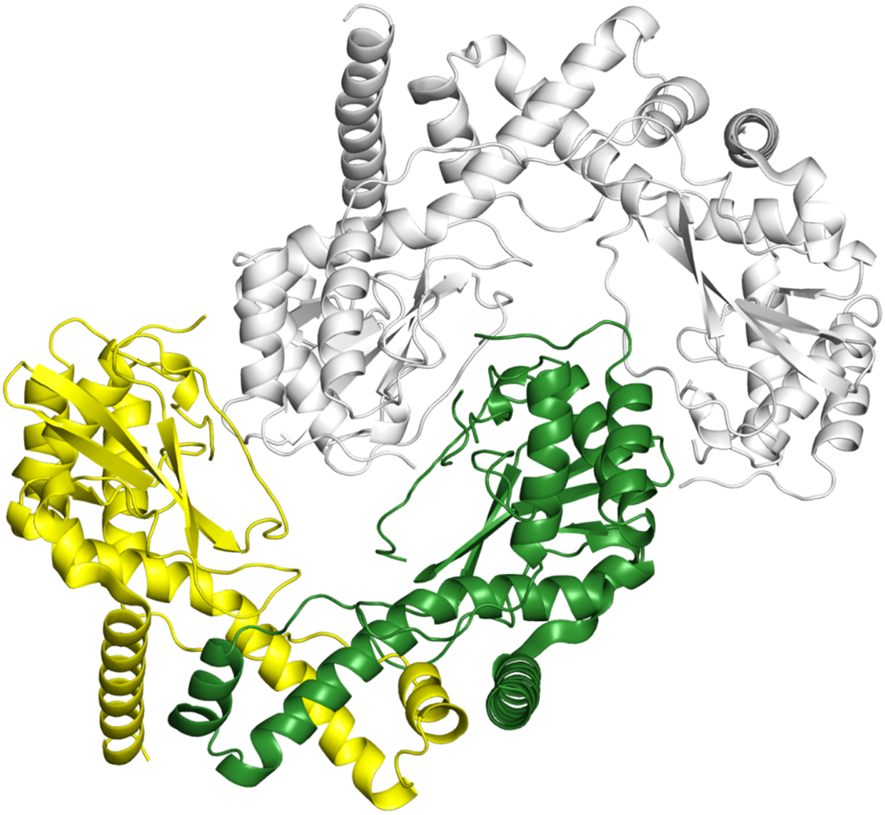

Fig. S5: Asymmetric unit of the AvaII crystals obtained in the absence of nucleic acids. The second molecule fills in the central cavity of the enzyme otherwise occupied by nucleic acid.

Fig. S6

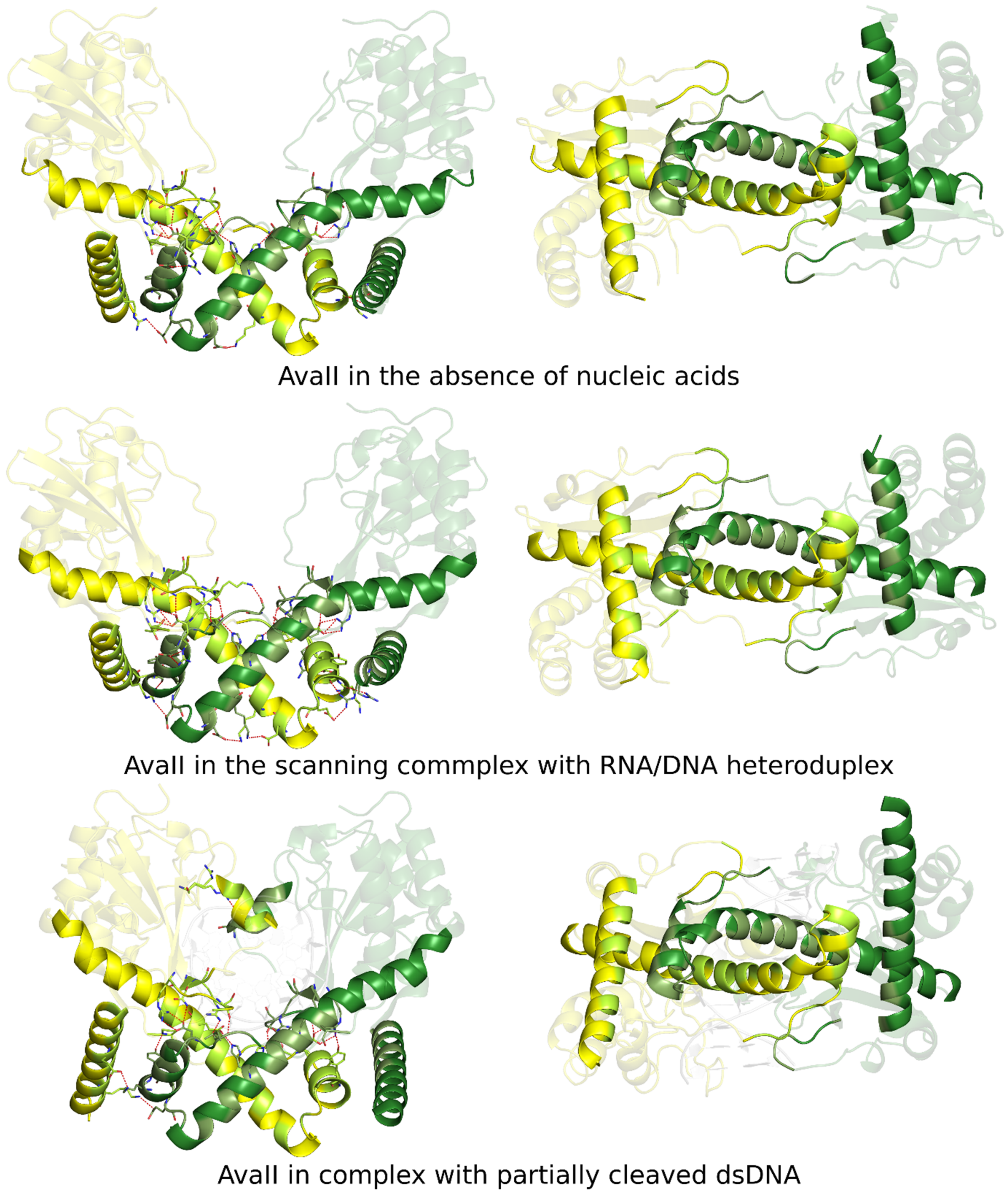

Fig. S6: Dimerization mode of AvaII in different forms. The dimerization mediating secondary structure elements are highlighted. The rest of the protein is shown in semi-transparent mode. The residues involved in the dimerization contacts are indicated as faint sticks and the possible hydrogen bonding interactions with faint dotted red lines.

Fig. S7

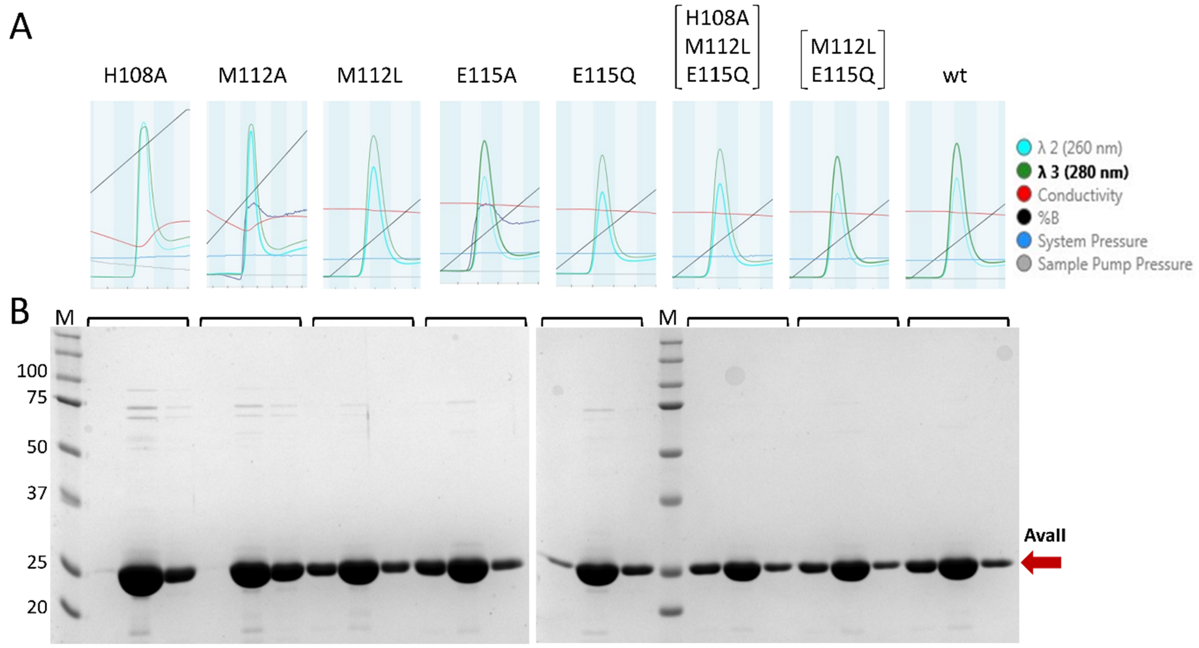

Fig. S7: Purification quality validation for AvaII variants by (A) gel filtration and (B) denaturing gel electrophoresis.

Fig. S8

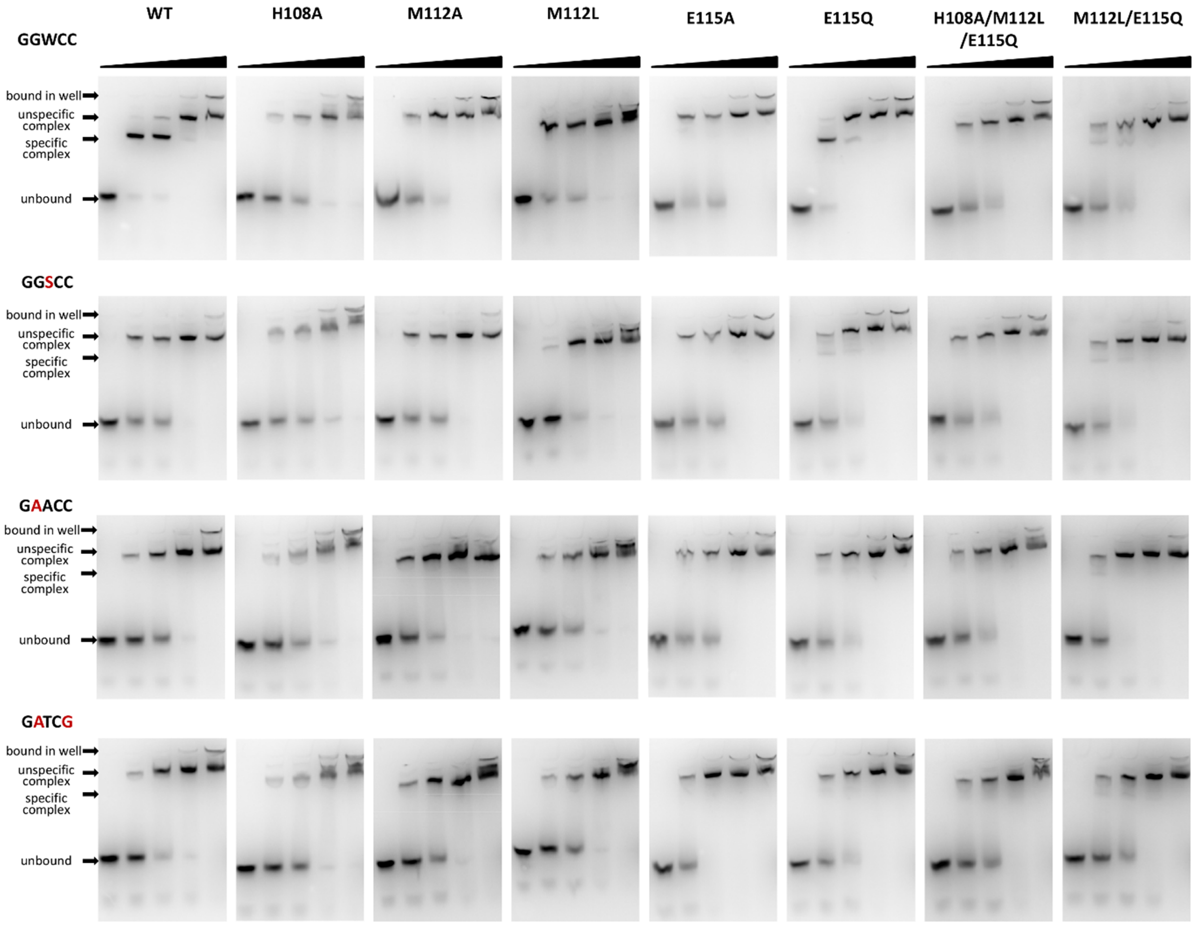

Fig. S8: Electrophoretic mobility shift assay (EMSA) of the interaction between wild type AvaII and its variants and dsDNA containing either a cognate (GGWCC) or miscognate (GGSCC, GAACC, GATCG) sites in the presence of Ca^{2+} ions. AvaII dimer concentration in each series was 0, 1, 2, 5 and 10 μM, dsDNA was 1 μM (29 bp).

Fig. S9

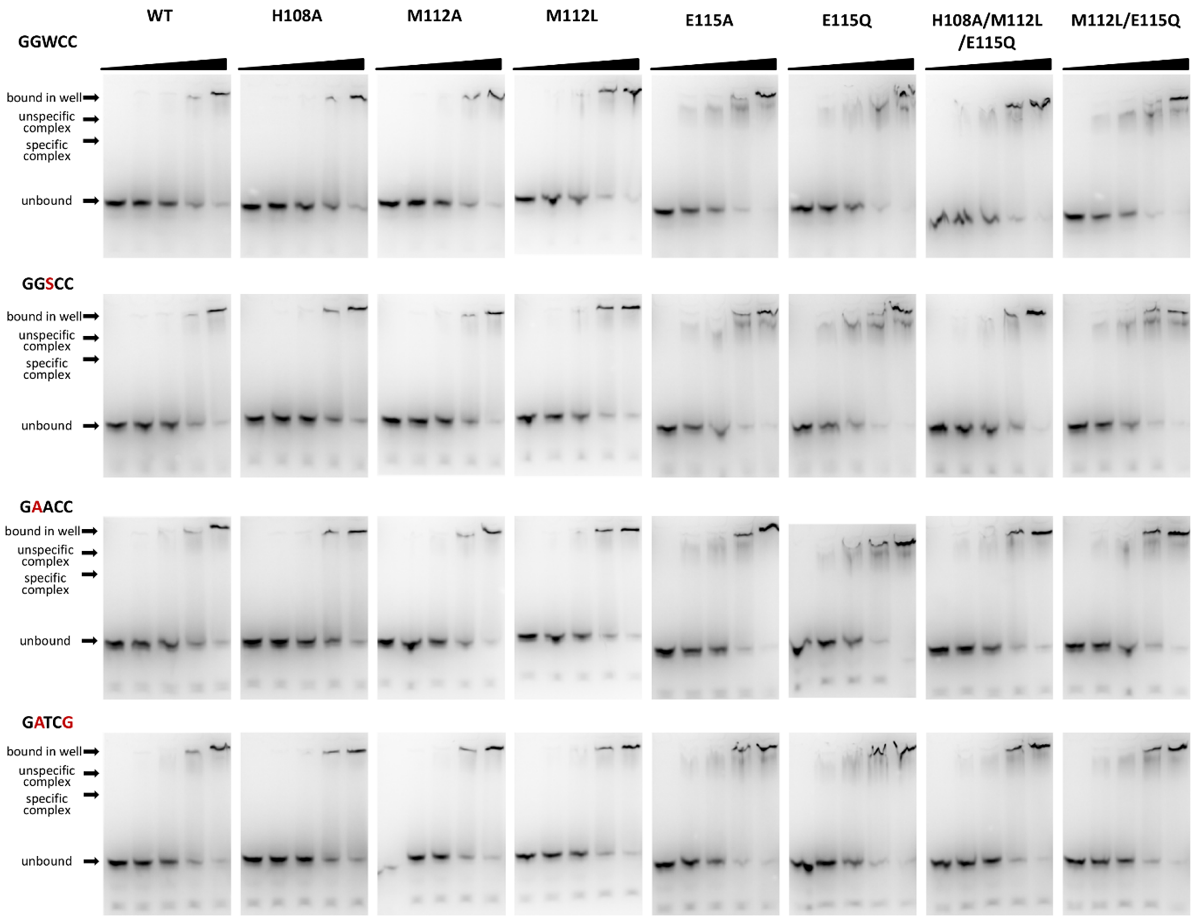

Fig. S9: Electrophoretic mobility shift assay (EMSA) of the interaction between wild type AvaII and its variants and dsDNA containing either a cognate (GGWCC) or miscognate (GGSCC, GAACC, and GATCG) sites in the presence <u>of EDTA</u>. AvaII dimer concentration in each series was 0, 1, 2, 5 and 10 µM, dsDNA was 1 µM (29 bp).

Fig. S10

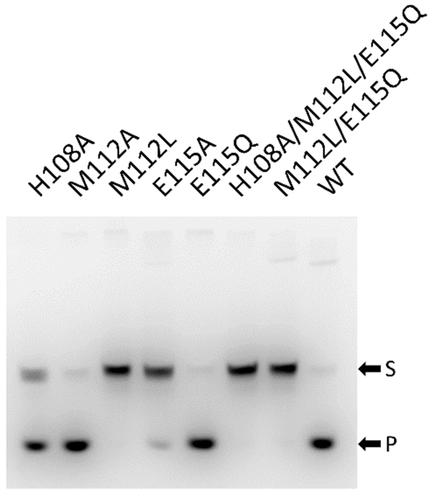

Fig. S10: Activity assay for AvaII variants. 0.1 µM AvaII was incubated with 1 µM dsDNA containing a single GGWCC cognate recognition site. S – substrate (29 bp), P – product (22 bp).

Fig. S11

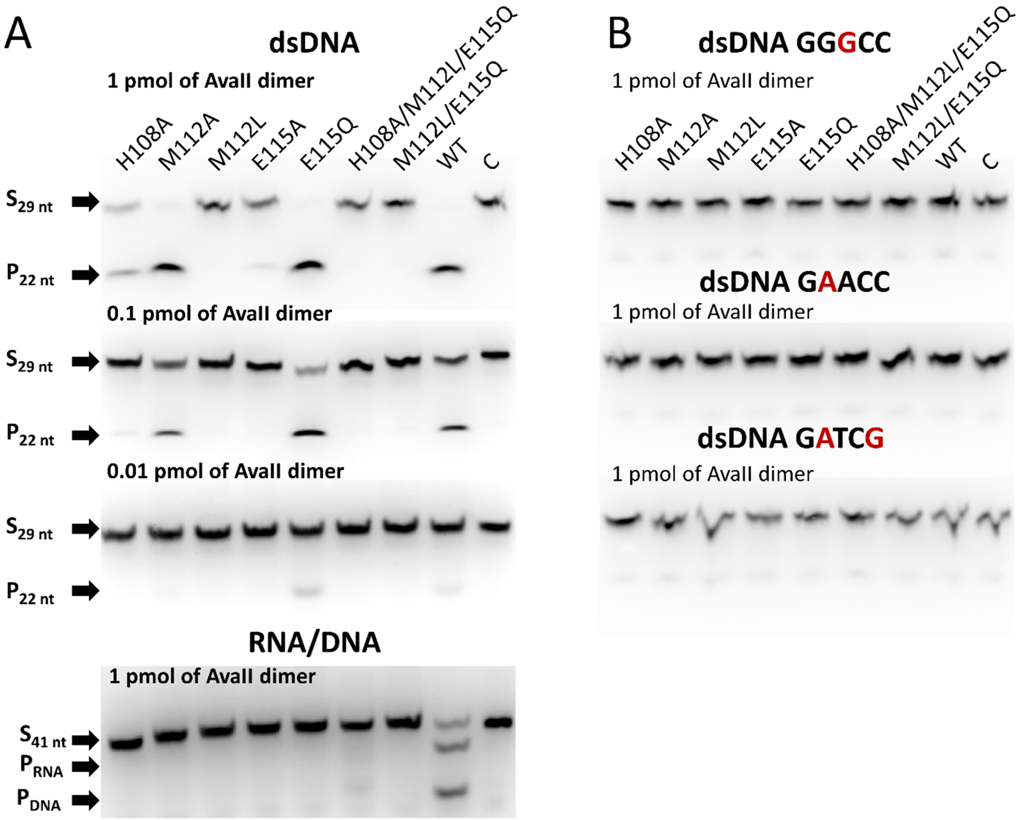

Fig. S11. Activity assay for AvaII variants on (A) dsDNA versus RNA/DNA hybrids and (B) dsDNA with alterations to AvaII target sequence. The cleavage assay was performed for 30 min at 37 °C. Only one strand was labelled with Cy5 in the dsDNA assays and 29 bp long substrate was used. Both strands were labelled in the RNA/DNA assay and 41 bp long substrate was used. The oligonucleotide sequences are listed in Table S1.

Fig. S12

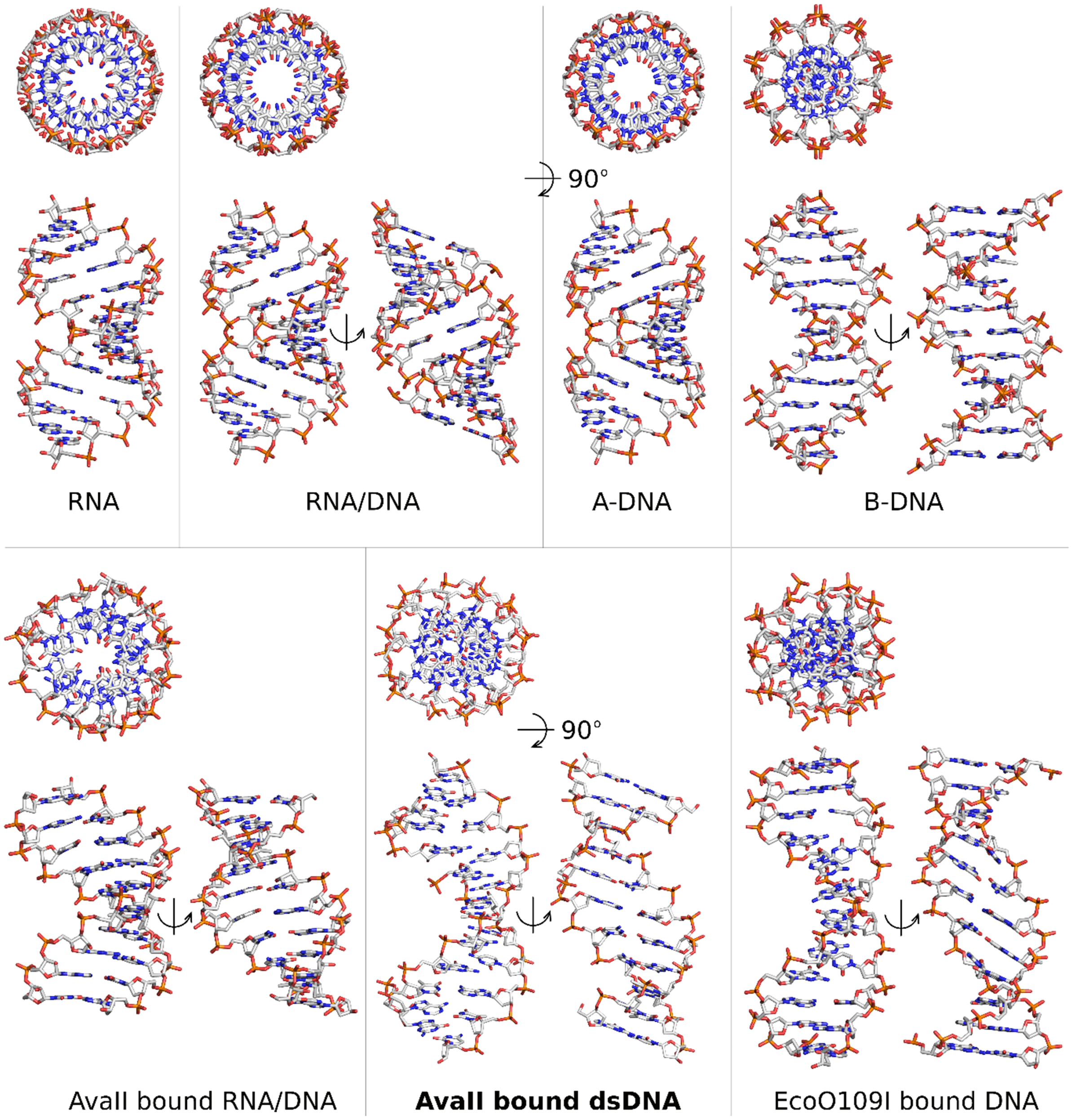

Fig. S12: Comparison of the idealized nucleic acid models with the oligoduplexes bound to AvaII and EcoO109I. Idealized duplexes were designed with the help of the 3DNA program (1). A homopolymer composed of A:dT base pairs was used to generate the idealized RNA/DNA. The AvaII and EcoO109I (2) bound dsDNA fragments resemble A-DNA in the center. The flanking parts of the oligoduplex revert to B-form in the EcoO109I complex but remain in between of A- and B-DNA in the AvaII-dsDNA structure. The conformation of the RNA/DNA hybrid in the complex with AvaII quite closely resembles the idealized RNA/DNA.

Fig. S13

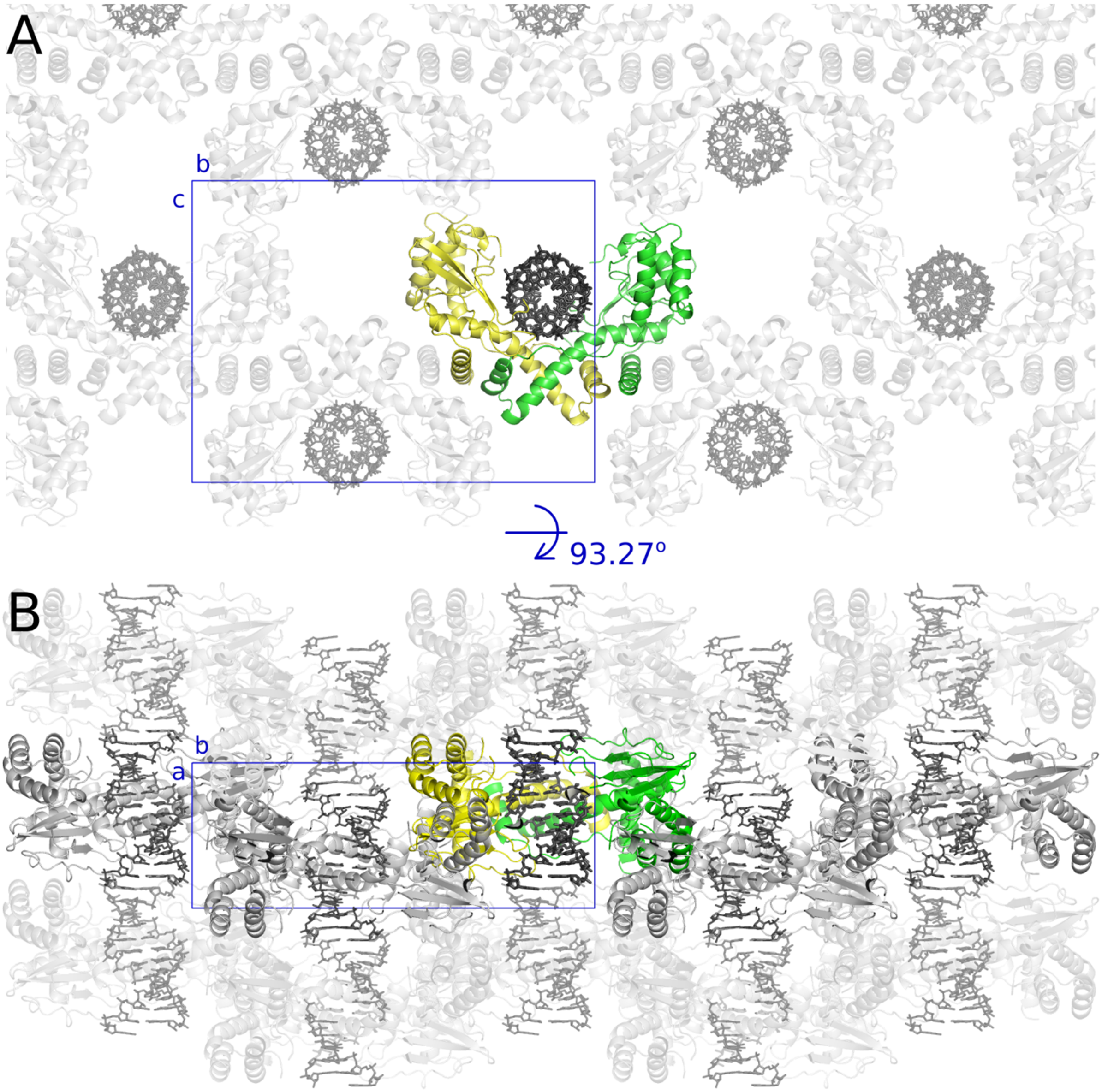

Fig. S13: Stacks of the AvaII dimers in the crystal, seen looking down the channels for dsDNA or RNA/DNA (panel A) or sidewise onto the channels for dsRNA or RNA/DNA (panel B). The content of the asymmetric unit of the crystal is shown in color (AvaII protomers in yellow and green, RNA/DNA heteroduplex in black). Other symmetry-equivalent protein-nucleic acid complexes are presented in gray. The blue box shows the monoclinic (and very nearly orthorhombic) unit cell.

Fig. S14

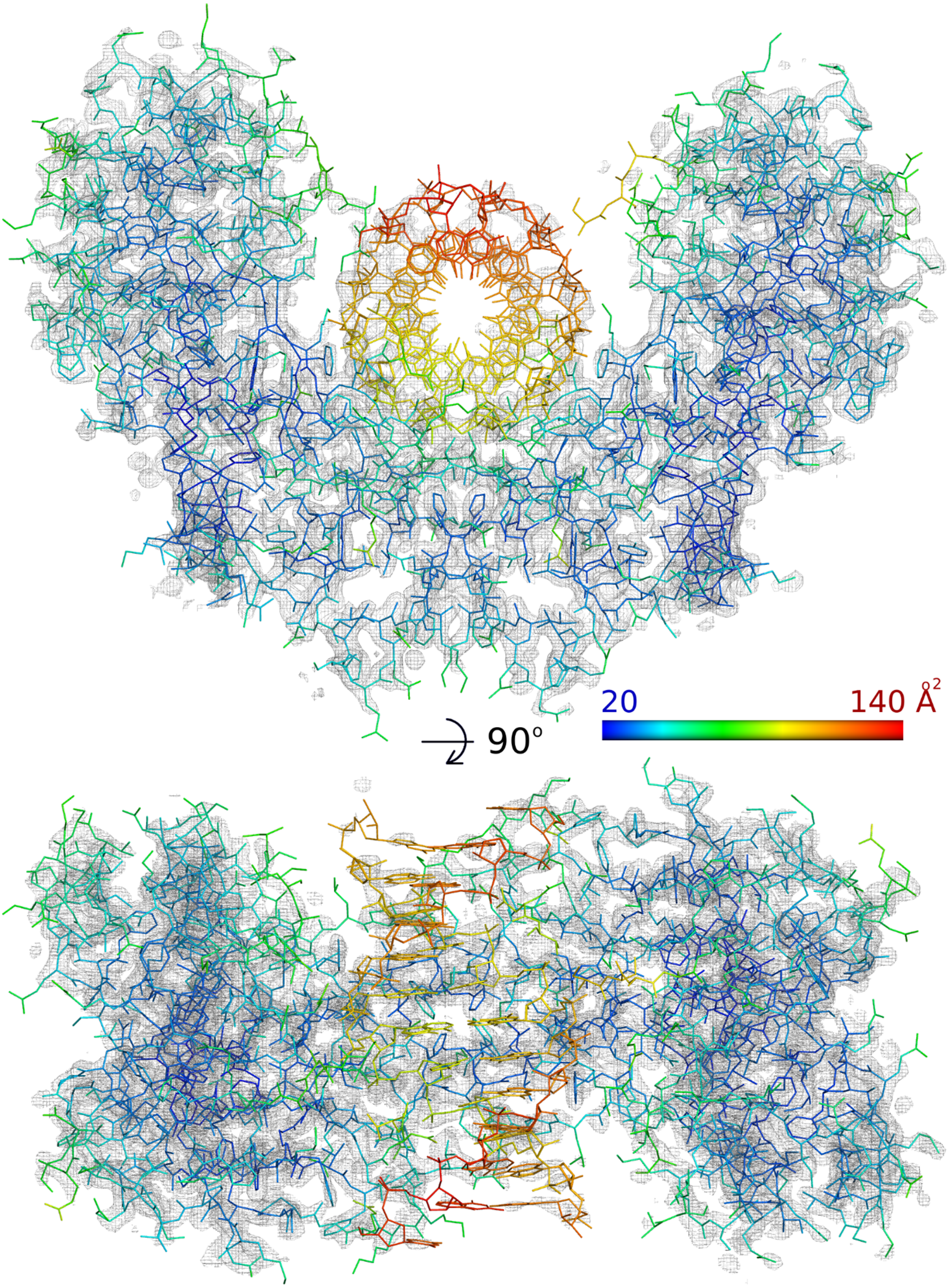

Fig. S14: AvaII scanning complex with RNA/DNA hybrid colored according to temperature factor, from low (blue) to high (red). The composite omit map was contoured at 1.5 rmsd and colored in gray.

Fig. S15

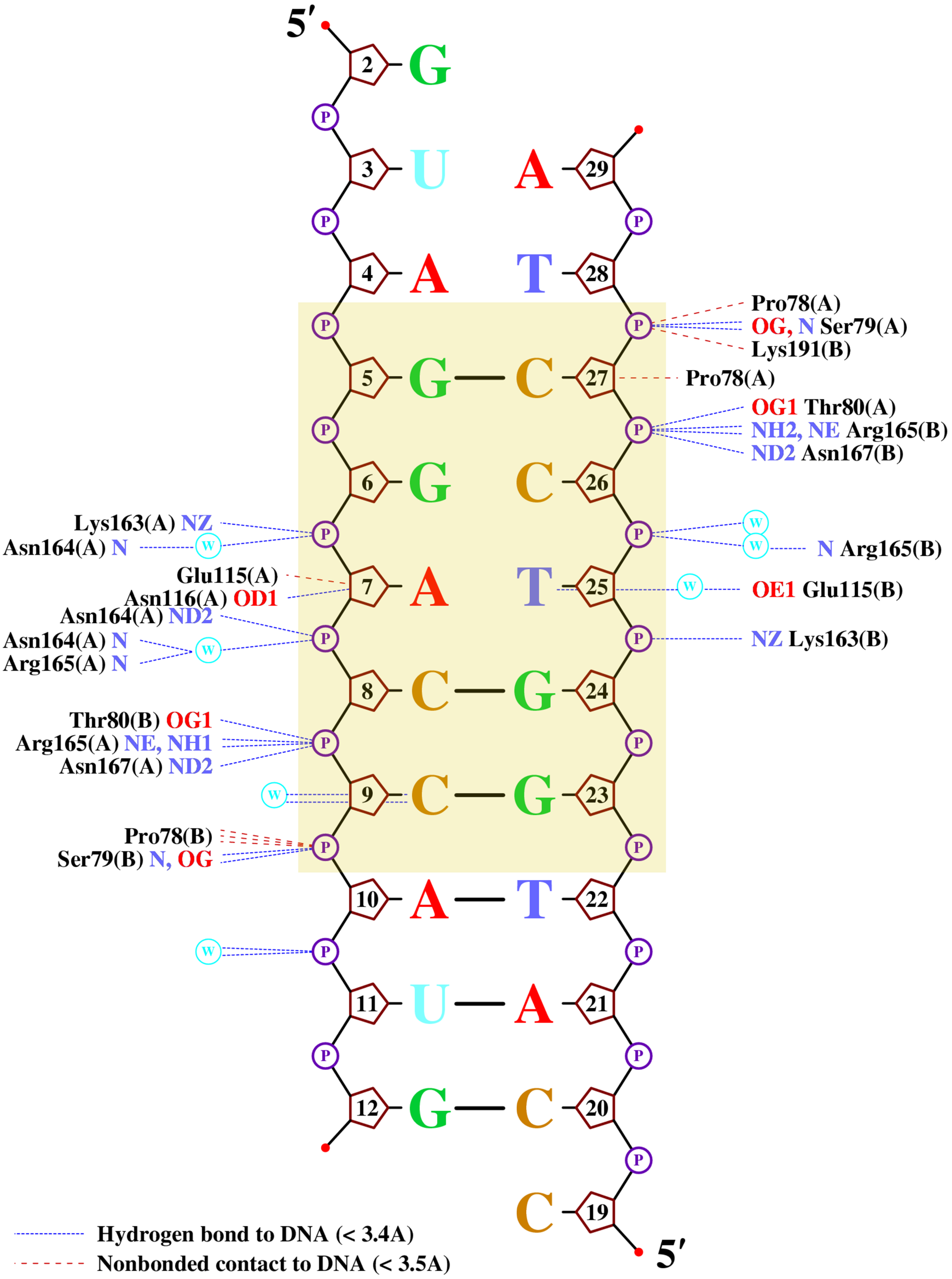

Fig. S15: Automatically assigned contacts between AvaII and the RNA/DNA heteroduplex. The NUCPLOT program (3) was used to depict one of the several possible heteroduplex binding modes that are compatible with the electron density. The RNA/DNA register was selected based on the position of the active site residues. The AvaII recognition sequence is shaded.

Fig. S16

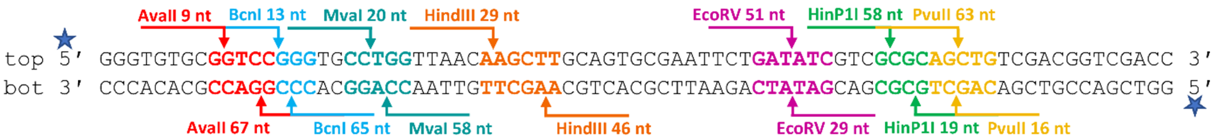

Fig. S16: The schematic view of the substrate used for the dsDNA and RNA/DNA cleavage tests.

Fig. S17

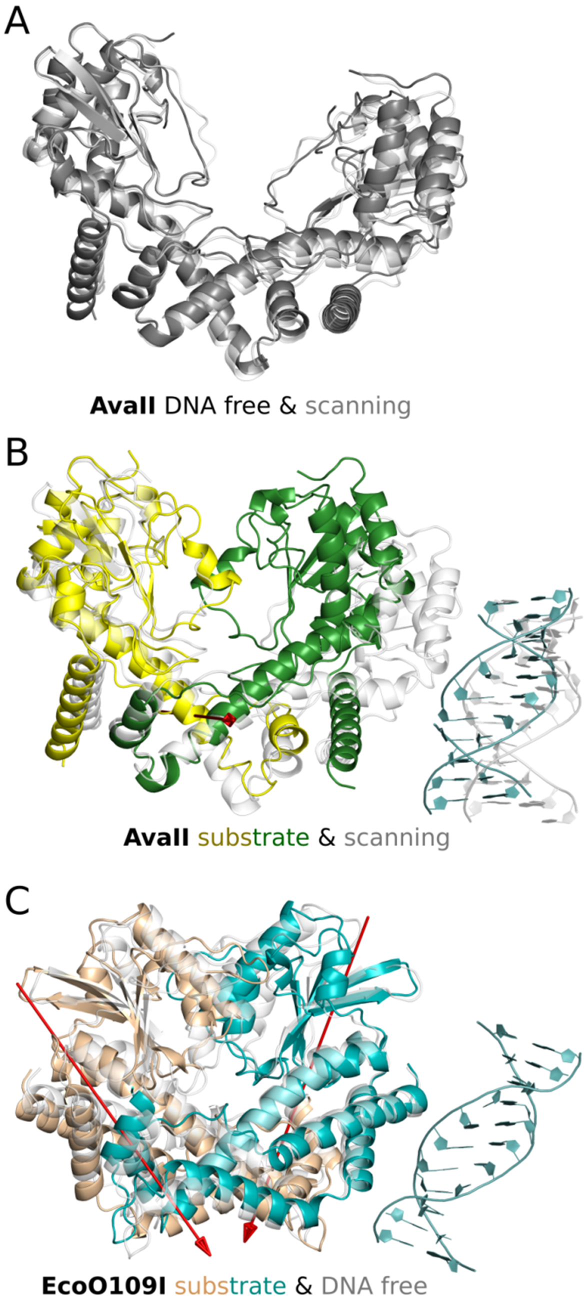

Fig. S17: Conversion from scanning to specific complex of AvaII and EcoO109I. The red arrows indicate the axes of rotation responsible for the closing of the enzymes predicted by DYNDOM (4).

Fig. S18

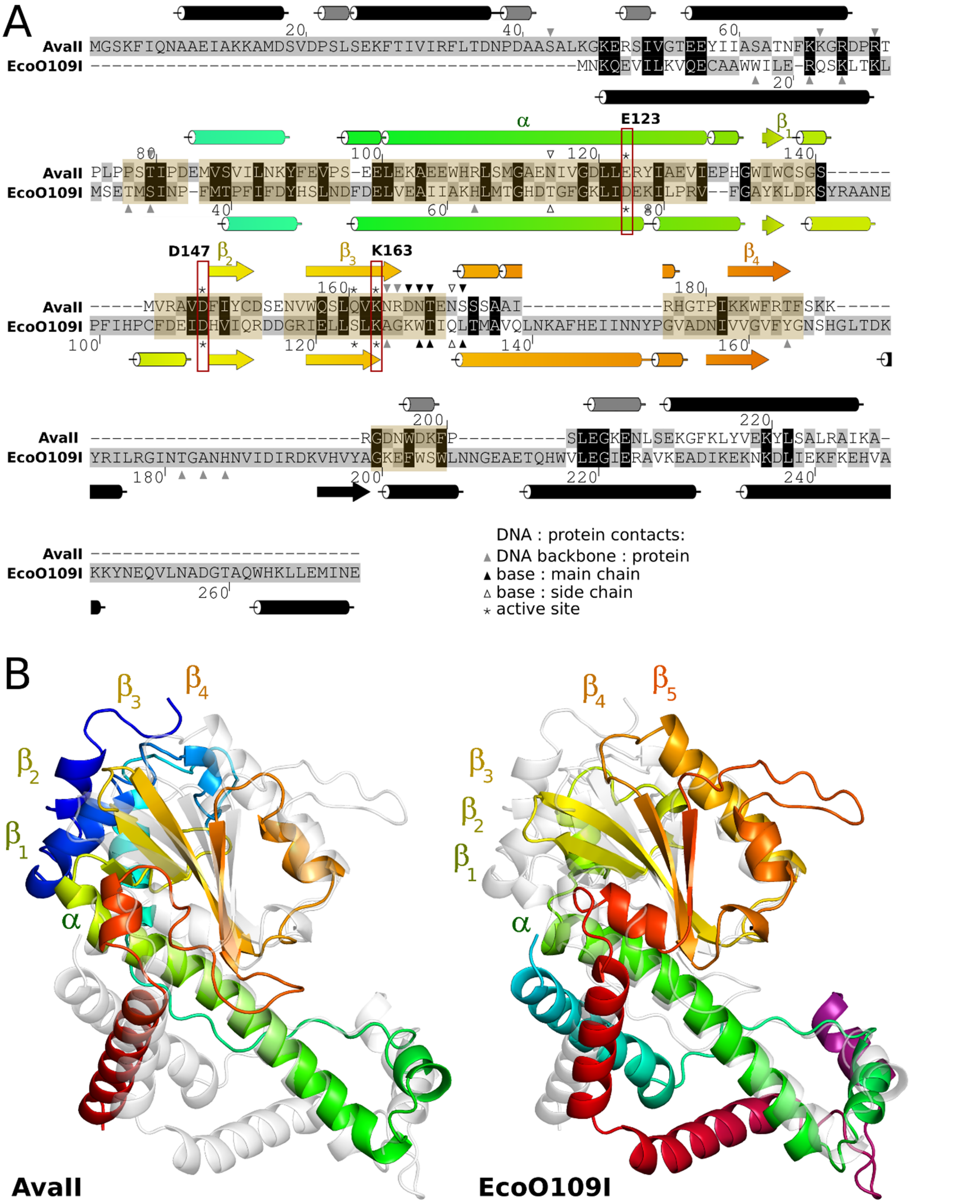

Fig. S18: AvaII and EcoO109I restriction endonucleases. (A) Combined structure and sequence based alignment. The structure based regions are indicated in sepia. Boxes mark residues with a proven role in catalysis. Residues that make contacts with DNA are indicated by triangles. The shared core region is colored as in panel B. **(B)** Location of secondary structure elements in AvaII and EcoO109I. The other enzyme is shown in faint grey color for comparison.

Fig. S19

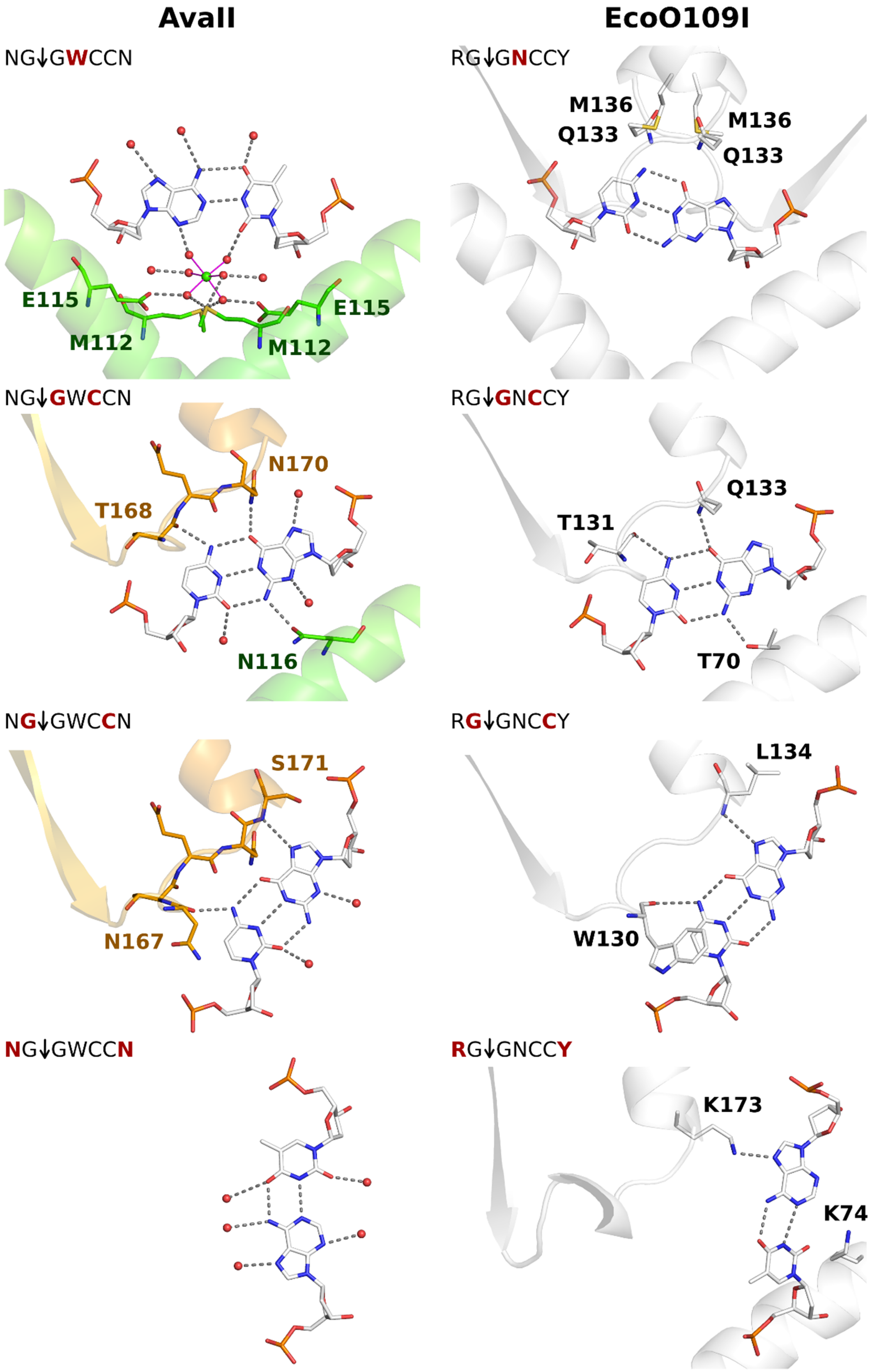

Fig. S19: AvaII and EcoO109I interactions with DNA bases.

Fig. S20

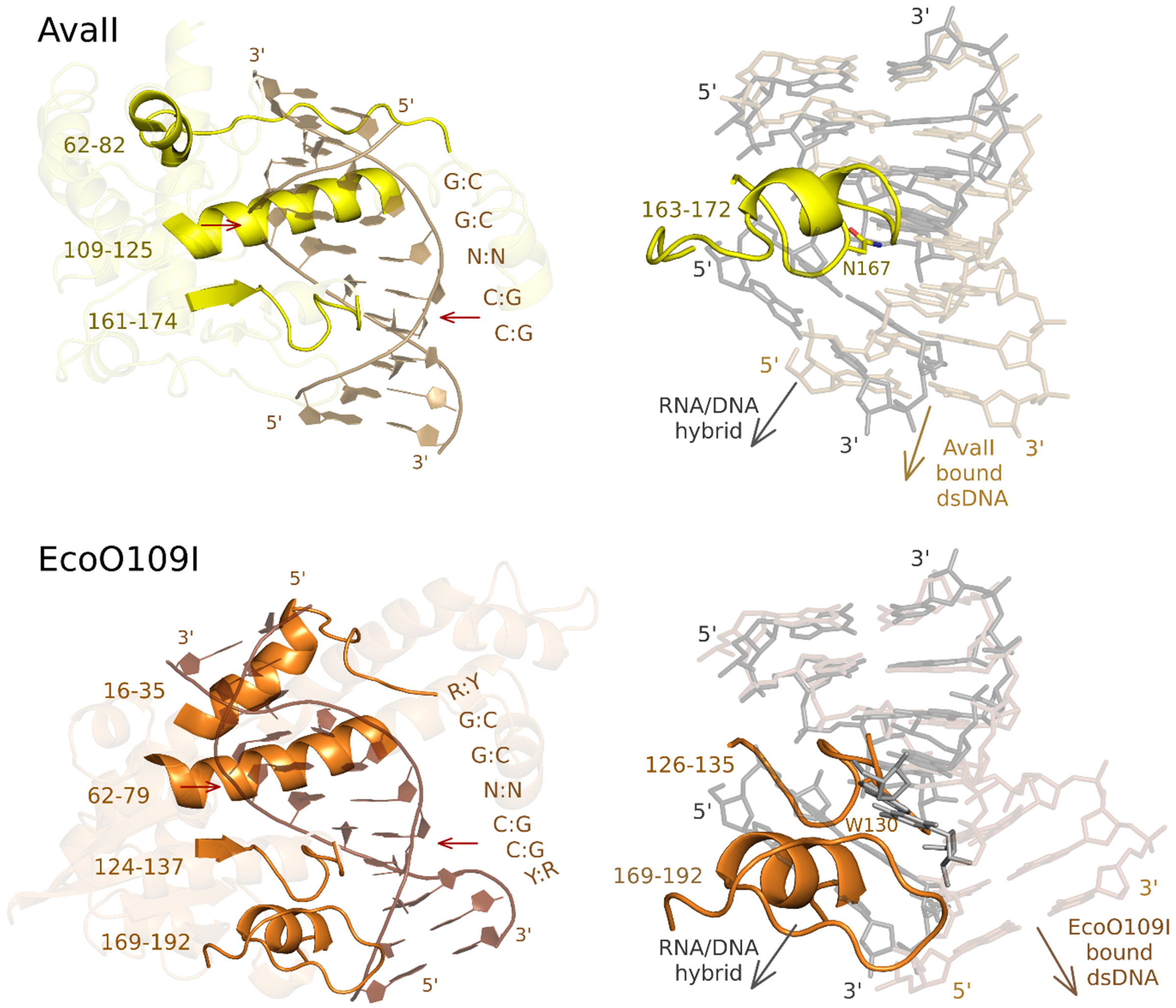

Fig. S20: Comparison of AvaII and EcoO109I DNA binding modes. The protein fragments that interact with central five GG(W/N)CC base pairs of the target DNA adopt similar conformation in AvaII (109-125, 161-174) and EcoO109I (16-35, 124-137). The fragments contacting the outer Y:R base pairs of the EcoO109I target are conformationally different (62-82 and 16-35 in the two enzymes) or absent in AvaII (169-192 in EcoO109I)(left). The Trp130 residue and 169-192 fragment that clash with the outer part of the RNA/DNA heteroduplex in EcoO109I are substituted by Asn167 and absent in AvaII (right).

Supplementary Table

Table S1: Oligonucleotides used in this study. AvaII target sites are indicated in bold.

Oligonucleotide sequence	Strand	Type	nt	3′ Cy5 label	Target	Purpose
5′ AAAGCUAAGC**GGACC**GAAUCGACUGCAUCGUCAUGAAAAAA 3′	top	DNA	41	+	GGWCC	AvaII activity assay
5′ AAAGCTAAGC**GGACC**GAATCGACTGCATCGTCATGAAAAAA 3′	top	RNA	41	+	GGWCC	"
5′ TTTTTTCATGACGATGCAGTCGATTC**GGTCC**GCTTAGCTTT 3′	bot	DNA	41	+	GGWCC	"
5′ GGAAGC**GGACC**AATCGACTGCATCGTCAG 3′	top	DNA	29	-	GGWCC	"
5′ CTGACGATGCAGTCGATT**GGTCC**GCTTCC 3′	bot	DNA	29	+	GGWCC	"
5′ GGAAGC**GGGCC**AATCGACTGCATCGTCAG 3′	top	DNA	29	-	GGSCC	"
5′ CTGACGATGCAGTCGATT**GGCCC**GCTTCC 3′	bot	DNA	29	+	GGSCC	"
5′ GGAAGC**GAACC**AATCGACTGCATCGTCAG 3′	top	DNA	29	-	GAACC	"
5′ CTGACGATGCAGTCGATT**GGTTC**GCTTCC 3′	bot	DNA	29	+	GGTTC	"
5′ GGAAGC**GATCG**AATCGACTGCATCGTCAG 3′	top	DNA	29	-	GATCG	"
5′ CTGACGATGCAGTCGATT**CGATC**GCTTCC 3′	bot	DNA	29	+	CGATC	"
5′ GGAAGC**GGACC**AAUCGACUGCAUCGUCAG 3′	top	RNA	29	-	GGWCC	"
5′ GGAAGC**GGGCC**AAUCGACUGCAUCGUCAG 3′	top	RNA	29	-	GGSCC	"
5′ GGAAGC**GAUCG**AAUCGACUGCAUCGUCAG 3′	top	RNA	29	-	-	"
5′ GGGTGTGC**GGTCC**GGGTGCCTGGTTAACAAGCTTGCAGTGCGAATTCTGATATCGTCGCGCAGCTGTCGACGGTCGACC 3′	top	DNA	79	-	GGWCC	RNA/DNA cleavage assay
3′ GGTCGACCGTCGACAGCTGCGCGACGATATCAGAATTCGCACTGCAAGCTTGTTAACCAGGCACCC**GGACC**GCACACCC 5′	bot	DNA	79	-	GGWCC	RNA/DNA cleavage assay

Table S2: Parameters of oligoduplexes bound to AvaII and EcoO109I in comparison with the ideal values. The characterization was performed with 3DNA (1). The target sequences are indicated in sepia. The A-like, B-like and unusual values are in red, blue and green, respectively. Only one conformation of two present in the crystals is shown. The pseudorotation angle (5) depiction was adopted from (6), other figures were adopted from the 3DNA manual (1). We have generously assumed the sugar puckers close to C2′-endo and C3′-endo to be B- and A-like, respectively, but the key puckers for the two DNA forms are C3'-endo, C2'-endo and C3'-exo. C↓CWGG AvaII target sequence was used as a ruler but the true oligoduplex in the EcoO109I-dsDNA structure (PDB: 1WTE) had a C:G pair in the center and complied with the RC↓CNGGY target of the enzyme (2).

A: Base and intra-strand parameters.

	Sugar conformation parameters								Intra-strand distances							
	Pseudorotation angle				Pucker				P - P				C1' - C1'			
**A-DNA**	**7.9**				**C3'-endo**, C2'-exo, C4'-exo[2]				**5.5**				**5.4**			
**B-DNA**	**153.9**				**C2'-endo, C3'-exo**, C1'-exo[2]				**6.6**				**4.9**			
DNA/RNA A/U	134.1/6.9				C1'-exo/C3'-endo				6.7/5.9				5.3/5.5			
T/A	14.6/13.8				C3'-endo/C3'-endo				5.7/5.7				5.5/5.5			
G/C	13.6/13.6				C3'-endo/C3'-endo				5.6/5.6				5.6/5.6			
I/C	139.9/12.6				C1'-exo/C3'-endo				6.9/6.2				5.3/5.4			
RNA	16				C3'-endo				5.8/5.7				5.5/5.4			
Base	**AvaII**		**EcoO109I**		**AvaII**		**EcoO109I**		**AvaII**		**EcoO109I**		**AvaII**		**EcoO109I**	
N	38	34	137	149	C4'-ex.	C3'-en.	C1'-ex.	C2'-en.	6.3	6.5	6	6.3	5.3	5.7	5.1	5.1
N	29	13	152	151	C3'-en.	C3'-en.	C2'-en.	C2'-en.	6	6.2	6.7	6.6	5.2	5.3	3.6	3.6
**G**	**9**	**10**	**21**	**22**	**C3'-en.**	**C3'-en.**	**C3'-en.**	**C3'-en.**	**7**	**7.8**	**6.6**	**6.9**	**5.6**	**5.6**	**5.6**	**5.8**
**G**	**15**	**9**	**13**	**13**	**C3'-en.**	**C3'-en.**	**C3'-en.**	**C3'-en.**	**5.6**	**4.7**	**5.6**	**5.7**	**5.5**	**5.4**	**5.4**	**5.5**
**A/T**	**18**	**132**	**25**	**3**	**C3'-en.**	**C3'-en.**	**C3'-en.**	**C3'-en.**	**6**	**6**	**6.4**	**6.3**	**6.2**	**6.3**	**6.5**	**6.3**
**C**	**156**	**158**	**173**	**176**	**C2'-en.**	**C2'-en.**	**C2'-en.**	**C2'-en.**	**6.9**	**6.8**	**7**	**6.9**	**5.5**	**5.6**	**6.1**	**6.1**
**C**	**146**	**127**	**210**	**213**	**C2'-en.**	**C1'-ex.**	**C3'-ex.**	**C3'-ex.**	**6.7**	**6.7**	**7.2**	**7.3**	**5.8**	**5.8**	**5.9**	**6**
N	147	162	197	166	C2'-en.	C2'-en.	C3'-ex.	C2'-en.	6.3	6.6	6.9	6.8	4.5	4.8	4.7	4.6
N	106	119	125	112	O4'-en.	C1'-ex.	C1'-ex.	C1'-ex.	7.2	6.6	6.9	7	5.4	5	4.4	4.9

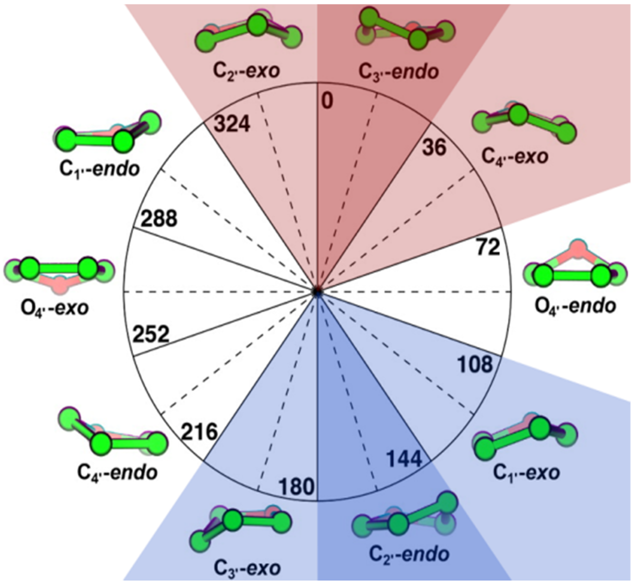

B: Base pair parameters

	Local base pair parameters				Global parameters based on C1'-C1' vectors and the helix axis			
	Propeller		Opening		Displacement		Angle	
**A-DNA**	**-10.5**		**-1.8**		**6.9**		**20.6**	
**B-DNA**	**-15.1**		**-1.6**		**1.9**		**-0.2**	
**DNA/RNA A/U**	-4.3		-1.7		5.9		11.8	
**DNA/RNA T/A**	10.5		-2.3		7.1		21.5	
**DNA/RNA G/C**	16.1		-1.3		7.2		19.2	
**DNA/RNA I/C**	-14.3		-1.8		4.6		12.1	
RNA	-2.08		-1.67		6.5		15.1	
	**AvaII**	**EcoO109I**	**AvaII**	**EcoO109I**	**AvaII**	**EcoO109I**	**AvaII**	**EcoO109I**
N-N	-15.8	-0.9	1.8	-4.0	7.0	8.4	1.8	-8.6
N-N	-6.8	-10.2	-1.2	-3.3	8.3	8.2	2.3	-2.4
**G-C**	**9.6**	**21.4**	**0.3**	**1.6**	**7.5**	**6.4**	**4.5**	**-1.0**
**G-C**	**0.2**	**2.5**	**0.2**	**0.7**	**7.6**	**6.6**	**6.6**	**-6.7**
**A-T**	**-11.9**	**-14.0**	**7.6**	**3.8**	**7.1**	**6.2**	**-0.1**	**-12.6**
**C-G**	**0.1**	**4.0**	**0.7**	**0.6**	**7.7**	**6.6**	**6.0**	**-6.5**
**C-G**	**9.5**	**18.2**	**1.4**	**-0.6**	**7.7**	**6.1**	**4.5**	**-1.3**
N-N	-0.2	-15.4	-0.5	-2.8	8.4	7.8	1.8	-1.7
N-N	-14.2	-2.2	1.5	-0.7	7.6	8.1	-0.3	-8.7
	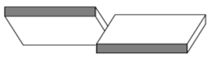		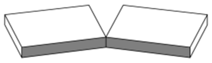				**A-DNA like**<br>**B-DNA like**<br>**unusual**	

C: Classification and parametrization of the base pair steps.

	Classification of dinucleotide steps		Double helix parameters				Local base-pair step parameters			
			Minor groove width		Major groove width		Slide		Roll	
**A-DNA**	**A**		**18.5**		**15.2**		**-1.4**		**12.4**	
**B-DNA**	**B**		**11.7**		**17.2**		**0.5**		**1.7**	
**DNA/RNA A/U**			16.1		17.6		-1.3		7.1	
**DNA/RNA T/A**	**A**		18.3		16.0		-1.7		10.8	
**DNA/RNA G/C**	**A**		18.7		16.0		-1.8		8.8	
**DNA/RNA I/C**			16.1		14.5		-0.6		9.0	
RNA	**A**		18.0		15.5		-1.5		8.6	
**step**	**AvaII**	**EcoO 109I**	**AvaII**	**EcoO 109I**	**AvaII**	**EcoO 109I**	**AvaII**	**EcoO 109I**	**AvaII**	**EcoO 109I**
NN/NN		B		15.0		17.8	-1.0	1.0	2.8	-2.3
NN/NN			15.1	13.6	18.1	21.8	-1.5	-0.7	-2.7	-33.2
**GG/CC**	**A**	**A**	**14.6**	**13.1**	**19.9**	**22.6**	**-1.9**	**-2.2**	**2.6**	**-4.2**
**GA/TC**	**A**	**A**	**15.1**	**14.1**	**20.5**	**22.6**	**-2.3**	**-2.4**	**9.6**	**10.1**
**AC/GT**	**A**	**A**	**15.1**	**14.3**	**20.8**	**22.8**	**-2.1**	**-2.1**	**8.8**	**8.0**
**CC/GG**	**A**	**A**	**14.6**	**13.3**	**20**	**22.8**	**-1.8**	**-2.1**	**1.3**	**-0.1**
NN/NN	A		15.6	13.6	17.6	21.8	-2.2	-0.1	-2.6	-29.4
NN/NN		B		14.6		17.5	-0.1	1.2	0.0	3.0
	**A-DNA like**<br>**B-DNA like**<br>**unusual**						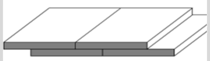		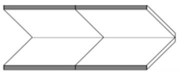	

D: Base pair position and orientation with respect to the helix axis.

	Local base-pair helical parameters							
	X-disp		Incl.		h-Rise		h-Twist	
**A-DNA**	**-4.5**		**22.7**		**2.5**		**32.6**	
**B-DNA**	**0.5**		**2.8**		**3.4**		**35.9**	
**DNA/RNA A/U**	-3.4		12.7		3.1		32.7	
**DNA/RNA T/A**	-4.7		19.5		2.6		32.7	
**DNA/RNA G/C**	-4.8		16.1		2.8		32.0	
**DNA/RNA I/C**	-2.2		14.8		3.1		36.0	
RNA	-4.1		15.5		2.8		32.7	
bp	**AvaII**	**EcoO109I**	**AvaII**	**EcoO109I**	**AvaII**	**EcoO109I**	**AvaII**	**EcoO109I**
NN/NN	-2.4	2.0	5.2	-3.8	3.2	3.2	31.4	34.5
NN/NN	-2.4	3.4	-5.4	-52.1	3.5	2.9	29.3	42.4
**GG/CC**	**-3.6**	**-2.7**	**4.3**	**-6.8**	**3.9**	**4.6**	**34.9**	**36.4**
**GA/TC**	**-5.7**	**-5.5**	**18.4**	**18.1**	**1.7**	**2.2**	**30.8**	**33.2**
**AC/GT**	**-5.8**	**-5.7**	**18.4**	**15.8**	**1.9**	**2.7**	**28.5**	**29.8**
**CC/GG**	**-3.3**	**-3.4**	**2.3**	**-0.1**	**3.9**	**4.5**	**34.7**	**36.6**
NN/NN	-4.2	3.1	-5.9	-42.6	3.7	2.7	26.0	44.3
NN/NN	-0.1	1.5	-0.1	5.2	3.4	3.4	38.8	32.9
**A-DNA like** **B-DNA like** **unusual**	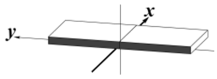		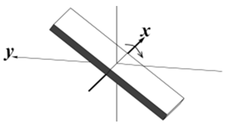		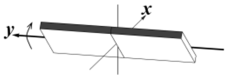		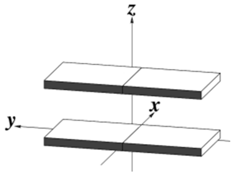	

References

1. Lu, X.J. and Olson, W.K. (2008) 3DNA: a versatile, integrated software system for the analysis, rebuilding and visualization of three-dimensional nucleic-acid structures. *Nat Protoc*, **3**, 1213-1227.
2. Hashimoto, H., Shimizu, T., Imasaki, T., Kato, M., Shichijo, N., Kita, K. and Sato, M. (2005) Crystal structures of type II restriction endonuclease EcoO109I and its complex with cognate DNA. *J Biol Chem*, **280**, 5605-5610.
3. Luscombe, N.M., Laskowski, R.A. and Thornton, J.M. (1997) NUCPLOT: a program to generate schematic diagrams of protein-nucleic acid interactions. *Nucleic Acids Res*, **25**, 4940-4945.
4. Hayward, S. and Berendsen, H.J. (1998) Systematic analysis of domain motions in proteins from conformational change: new results on citrate synthase and T4 lysozyme. *Proteins*, **30**, 144-154.
5. Altona, C., Sundaralingam, M. (1972) Conformational analysis of the sugar ring in nucleosides and nucleotides. A new description using the concept of pseudorotation. *J Am Chem Soc*, **94**, 8205–8212.
6. Li, L., Szostak, J.W. (2014) The free energy landscape of pseudorotation in 3'-5' and 2'-5' linked nucleic acids. *J Am Chem Soc,* **136**, 2858-2865.
